# AI-navigated shoulder injection: precision, real-time learning and clinical translation

**DOI:** 10.3389/frai.2026.1727704

**Published:** 2026-03-06

**Authors:** Hua Li, Xiaodan Huang, Ming Zhao, Yanxin Cheng

**Affiliations:** 1 Department of Ultrasound, Third Hospital of Hebei Medical University, Shijiazhuang, Hebei, China; 2 Department of Orthopedics, Third Hospital of Hebei Medical University, Shijiazhuang, Hebei, China; 3 Department of Pain, Third Hospital of Hebei Medical University, Shijiazhuang, Hebei, China

**Keywords:** AI-navigated navigation technology, artificial intelligence, regulation, shoulder joint injection, ultrasound guidance

## Abstract

This review focuses on artificial intelligence (AI)-guided ultrasound-guided shoulder joint injections. We systematically retrieved relevant studies from PubMed, Embase, Cochrane Library, IEEE Xplore, and Web of Science (1996 ~ 2025). Literature was screened based on predefined inclusion/exclusion criteria, and evaluated AI technologies using core metrics including anatomical segmentation accuracy (Dice similarity coefficient), first-pass puncture success rate, and clinical outcome indicators (Visual analogue scale scores, VAS; American Shoulder and Elbow Surgeons, ASES scores). It explores the technical principles of AI medical image processing (segmentation, detection, tracking, reconstruction) and deep learning algorithms for shoulder anatomy, addressing limitations of traditional ultrasound guidance through AI-enabled precision targeting and real-time learning. Clinical applications, technological advancements, ethical controversies, and regulatory pathways are summarized. Key findings confirm AI enhances injection accuracy, first-pass success rates, and patient outcomes. This work provides a concise, evidence-based reference for clinicians and researchers, highlighting the paradigm shift of AI in optimizing shoulder injection therapy.

## Introduction

The ultrasound-guided injection technique has gradually emerged as an important alternative to traditional blind injection methods in modern medical practice. This approach offers distinct advantages in enhancing injection accuracy, reducing the need for repeat injections, and alleviating both the financial and psychological burdens on patients. High-quality evidence firmly establishes ultrasound-guided injections as a superior alternative to traditional blind techniques for musculoskeletal interventions, including shoulder joint injections (Karaahmet et al., 2017; Wu et al., 2024; Aly et al., 2015). A meta-analysis of 15 comparative studies (*n* = 1,287 patients) confirmed that ultrasound-guided injections achieved 25 ~ 30% higher accuracy across most shoulder girdle sites and significantly better clinical outcomes (e.g., reduced pain, improved function) compared to landmark-guided methods (Aly et al., 2015). A prospective randomized controlled trial (RCT) further validated this superiority: in botulinum toxin injections for glabellar wrinkles, ultrasound-guided delivery yielded 40% greater wrinkle reduction and 35% longer duration of effect than blind injection (Wu et al., 2024), a principle translatable to shoulder interventions where precise targeting is critical. For severe carpal tunnel syndrome, another condition requiring precise soft-tissue injection, a randomized comparative study showed ultrasound-guided steroid injections improved hand function by 32% at 3 months, compared to 18% with blind injection (Karaahmet et al., 2017). These findings, anchored in meta-analyses and RCTs, confirm that ultrasound-guided injection’s core advantage lies in precise targeting of pathological sites, directly enhancing therapeutic efficacy. Beyond superior efficacy, ultrasound-guided injections deliver tangible long-term clinical and economic benefits. A large cohort study (*n* = 523) of carpal tunnel syndrome patients demonstrated that ultrasound-guided steroid injections reduced retreatment rates by 30% over 12 months, translating to lower long-term medical costs despite higher initial equipment investment (Evers et al., 2017). For patients with inflammatory chronic arthritis, a randomized comparative study confirmed that ultrasound-guided local corticosteroid injections reduced VAS scores by 2.1 points at 6 weeks, nearly double the reduction achieved with blind injection (Gutierrez et al., 2016). This pain relief advantage is particularly relevant for shoulder disorders, as a systematic review and meta-analysis of 8 RCTs showed ultrasound-guided intra-articular/periarticular injections for shoulder pathologies (e.g., rotator cuff tendinopathy) reduced VAS scores by 1.8 ~ 2.3 points at 2 ~ 6 weeks post-injection (Huang et al., 2015). Even in specialized populations, such as paraplegic spinal cord injury patients with rotator cuff tendinopathy, a single-blind RCT found ultrasound-guided subacromial bursa injections improved shoulder mobility by 28% compared to blind injection (Azadvari et al., 2021). Collectively, these data confirm that ultrasound guidance’s precision translates to reduced retreatment, enhanced pain relief, and broader applicability across patient groups, justifying its adoption in shoulder joint injection practice.

Ultrasound-guided technology has undergone significant evolution in the application of shoulder joint injections. In early stages, it was primarily used for diagnosing shoulder joint disorders, such as assisting in the identification of conditions like rotator cuff tears and bursitis through ultrasound imaging (Bergman et al., 2025). With technological advancements, it has gradually been applied to shoulder joint injections to enhance injection accuracy and therapeutic efficacy. Ultrasound-guided steroid -injection has become a common treatment for calcific tendinitis (Hong et al., 2015). However, the technology still suffers from bottlenecks. On one side, the clarity and accuracy of ultrasound imaging are limited for certain complex shoulder joint anatomical structures, such as the subscapularis tendon and deep regions of the glenohumeral joint, making it difficult to precisely guide the injection needle to the target location. On the other side, in actual practice, patient individual differences, such as body type and shoulder anatomical variations, increase the difficulty of ultrasound guidance and reduce the success rate of injections. For instance, obese patients have thicker shoulder fat layers, which attenuate ultrasound signals, compromising image quality and consequently affecting the precision of ultrasound guidance (Diplock et al., 2023). Additionally, traditional ultrasound guidance relies heavily on the operator’s experience and skill, with significant variations in proficiency among different practitioners, leading to inconsistent injection outcomes. The novel breakthrough is required for prospective clinical practice.

The shift from static imaging to dynamic navigation represents a significant advancement in the application of ultrasound-guided techniques for shoulder joint injections. Static ultrasound images provide some anatomical information, but they have limitations during real-time guided injections. It has been demonstrated that ultrasound-guided shoulder belt injections exhibit higher accuracy at most injection sites compared to injections guided by anatomical landmarks. However, in the subacromial space, the difference in accuracy between the two methods is not significant (Aly et al., 2015). The insignificant difference may be due to the inability of static images to reflect in real time the positional changes of the injection needle during the dynamic process, as well as the real-time movement of surrounding tissues. Dynamic navigation aims to address this issue by providing operators with more precise guidance through real-time tracking of the injection needle and surrounding tissue movements. Achieving high-quality dynamic navigation confronts technical challenges. For one thing, the limited frame rate of ultrasound images struggles to meet the demands of real-time, high-resolution dynamic display (Boni et al., 2017; Grondin et al., 2015). This makes it difficult to promptly capture needle position and surrounding tissue changes during rapid injections or when tissue movement is fast. Then, the accuracy of dynamic navigation systems is further compromised by factors such as ultrasound signal interference and image registration errors (Diplock et al., 2023; Tamborrini et al., 2024b). These limitations hinder the effective transition from static imaging to dynamic navigation, thereby impeding the advancement of ultrasound-guided techniques in shoulder joint injections toward greater precision and efficiency (Rickmann et al., 2020).

**Figure 1:**
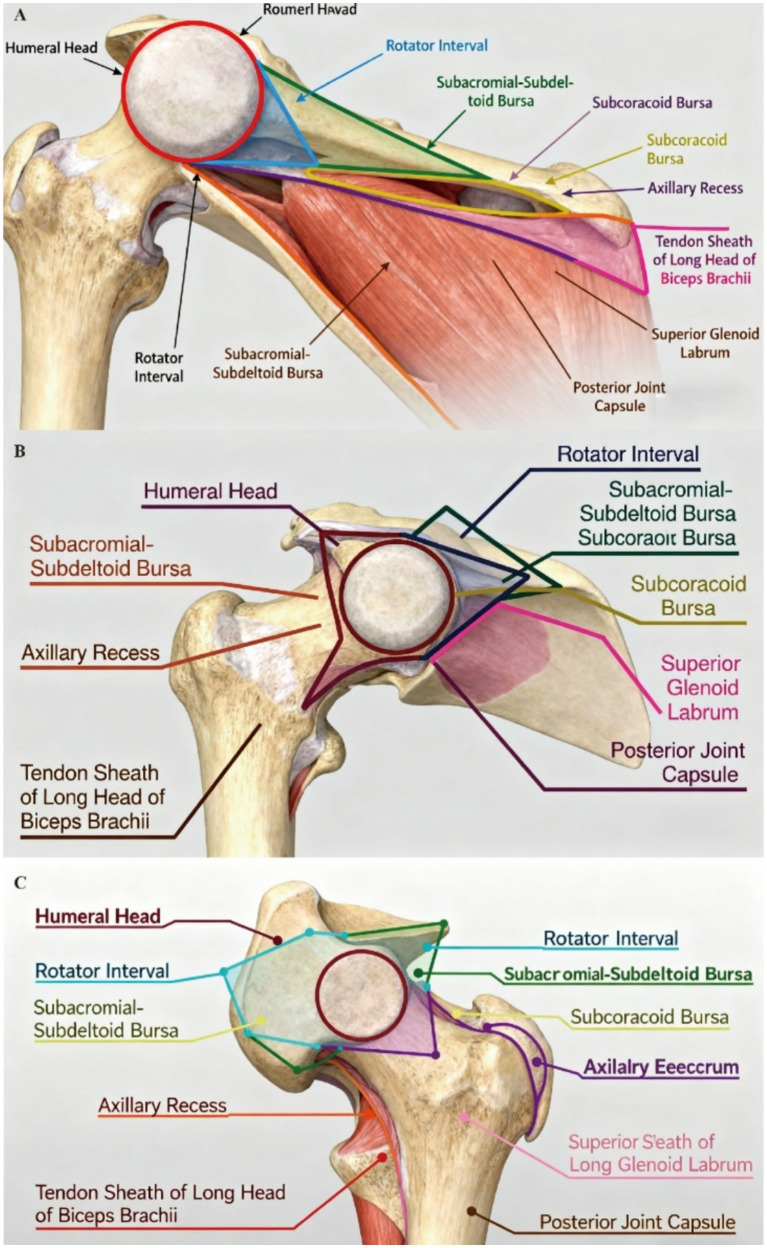
Anatomical diagrams of the shoulder joint. **(A)** (coronal view): anterolateral perspective showing the humeral head with the rotator interval above (containing the long head of biceps tendon), subacromial-subdeltoid bursa, subcoracoid bursa, axillary recess, and intact posterior capsule, critical for AI identifying superficial target sites (e.g., subacromial bursa). **(B)** (sagittal view): Lateral perspective demonstrating the “sandwich” arrangement of bursae (subacromial-subdeltoid most superior) and tendon sheath around the rotator cuff, illustrating the anatomical complexity that challenges static ultrasound guidance (resolved by AI dynamic tracking). **(C)** (axial view): Cranial-to-caudal view with the humeral head centered, triangular rotator interval, and semicircular bursal system, providing spatial coordinates for AI 3D reconstruction and needle path optimization.

The appearance of AI-navigated ultrasound has brought a dual revolution to shoulder joint injections: precise targeting and real-time learning. This innovation enables deep analysis of ultrasound images, accurately identifies key anatomical structures of the shoulder joint (e.g., the rotator cuff and glenohumeral joint), and thereby guides the injection needle to reach the target location precisely. AI has proven to construct precise anatomical models through deep learning of extensive shoulder joint ultrasound images, providing more accurate path planning for injections (Dubey et al., 2025). The anatomy of the shoulder joint was shown in Figure 1. Specifically, Figure 1A (coronal view) clarifies the spatial relationship between the humeral head, rotator interval, and subacromial-subdeltoid bursa, key structures for AI segmentation and path planning. Figure 1B (sagittal view) demonstrates the “sandwich” arrangement of bursae and tendons, explaining why static ultrasound struggles to visualize dynamic needle movement in the subacromial space (addressed by AI real-time tracking). Figure 1C (axial view) outlines the semicircular bursal system and posterior joint capsule, providing anatomical references for AI to avoid critical structures during injection.

Real-time learning represents another major breakthrough in AI-navigated ultrasound navigation. Traditional navigation systems typically rely on predefined models and algorithms, lacking the ability to adjust in real time based on actual operating conditions. AI-powered ultrasound navigation, instead, collects data in real time during the injection process, needle position, surrounding tissue feedback, and more, and uses machine learning algorithms to continuously update and optimize the model. In particle therapy planning, for instance, deep learning-based voxel sampling models can reduce computational load based on real-time feedback, thereby enhancing planning efficiency while maintaining plan quality (Quarz et al., 2024). The capability allows AI-navigated ultrasound navigation to adapt to individual patient variations and complex operating environments, continuously enhancing injection precision and safety, thereby revolutionizing shoulder joint injections.

Notably, this review distinguishes itself from existing publications in three key aspects, filling critical gaps in the current literature. First, comprehensive integration of technical principles and clinical translation: Most previous reviews either focus on ultrasound-guided shoulder injection techniques alone or discuss single AI algorithms for musculoskeletal image processing in isolation. In contrast, this review systematically bridges AI medical image processing workflows (segmentation, detection, real-time tracking, 3D reconstruction), real-time learning closed-loop mechanisms, and practical clinical issues (regulatory approval pathways, ethical controversies, and patient-centric impacts), forming a complete “technology-clinic” research chain. Second, establishment of a standardized AI tool evaluation framework: No prior review has proposed a structured evaluation system for AI-navigated shoulder injection tools. This review constructs a multi-dimensional criterion system covering accuracy, generalizability, safety, clinical integration, and regulatory status, providing a unified reference for clinicians to select appropriate tools and for researchers to optimize algorithm design. Third, focus on underrepresented populations and cross-joint translation potential: Existing reviews rarely address the performance limitations of AI systems in special patient groups (e.g., BMI > 35 individuals, patients with anatomical variations) or the migration of AI-navigated injection technology to other major joints (hip, knee, ankle). This review systematically analyzes these gaps, proposes targeted solutions, and discusses the cross-joint application prospects, laying a foundation for the broader application of this technology.

## Materials and methods

### Literature search strategy

A systematic literature search was conducted across five electronic databases, including PubMed, Embase, Cochrane Library, IEEE Xplore, and Web of Science, to identify studies relevant to AI-navigated ultrasound-guided shoulder joint injections. The search combined the following keywords and their synonyms: “artificial intelligence,” “AI navigation,” “ultrasound guidance,” “shoulder joint injection,” “rotator cuff injection,” “musculoskeletal intervention,” and “precision injection.” The literature search encompasses studies from 1996 to 2025, ensuring the inclusion of timely, relevant, and comprehensive research.

### Inclusion and exclusion criteria

Inclusion criteria: (1) Studies focusing on the technical principles, clinical applications, or efficacy evaluation of AI-navigated ultrasound-guided shoulder joint injections. (2) Studies providing quantitative original data on key clinical outcomes, including first-pass puncture success rate, Visual Analogue Scale (VAS) score reduction, American Shoulder and Elbow Surgeons (ASES) score improvement, and re-injection rate. (3) Study types covering randomized controlled trials (RCTs), cohort studies, case–control studies, technical validation studies, and high-quality systematic reviews/meta-analyses. (4) Publications written in English to ensure data extraction consistency.

Exclusion criteria: (1) Animal experiments, *in vitro* studies, and case reports without quantitative efficacy data. (2) Studies focusing on ultrasound-guided injections for joints other than the shoulder or non-injection-related musculoskeletal interventions. (3) Conference abstracts, dissertations, and studies with incomplete or unextractable key outcome indicators. (4) Studies with low methodological quality, as assessed by the evaluation scales described below.

### Study selection process

Two independent reviewers screened the literature in two consecutive stages: title and abstract screening, followed by full-text evaluation. In the first stage, irrelevant studies were excluded based on the inclusion and exclusion criteria. In the second stage, the full texts of potentially eligible studies were retrieved for detailed assessment. Any discrepancies between the two reviewers were resolved through discussion with a third senior reviewer. The PRISMA flow was prepared to illustrate the detailed literature screening process, including the number of retrieved, excluded, and finally included studies at each stage (Table 1).

**Table 1:** The PRISMA flow.

PRISMA flow attributes
Study identification
Electronic database searching (PubMed, Embase, Cochrane, IEEE Xplore, Web of Science)
Total records identified: *n* = 826
Additional records identified via other sources: *n* = 0
Total records screened: *n* = 826
Study screening
Records after duplicates removed: *n* = 668
Records excluded after title/abstract screening: *n* = 592
Reasons for exclusion (title/abstract):
Non-English publications: *n* = 133
Unrelated research (non-shoulder joint injection/non-AI navigation): *n* = 149
No quantitative efficacy data: *n* = 181
Conference abstracts/theses (unpublished literature): *n* = 129
Full-text articles assessed for eligibility: *n* = 76
Full-text articles excluded: *n* = 3
Reasons for exclusion (full-text):
Low methodological quality (NOS/AMSTAR 2 criteria not met): *n* = 1
Incomplete key outcome indicators (unextractable DSC/VAS/ASES/success rate): *n* = 1
Animal experiments/*in vitro* studies/case reports without quantitative data: *n* = 1
Study eligibility
Total studies included in the qualitative synthesis: *n* = 73

### AI tool selection criteria

To ensure the representativeness and clinical relevance of the AI tools evaluated in this review, the following selection criteria were applied: (1) The AI tool covers the core technical modules for shoulder joint injection navigation, including anatomical segmentation, lesion detection, needle trajectory tracking, and 3D reconstruction. (2) The AI tool has undergone clinical validation in human studies or obtained regulatory approval from authorities such as the National Medical Products Administration (NMPA), U.S. Food and Drug Administration (FDA), or European Union (EU) Conformité Européenne (CE). (3) The performance data of the AI tool have been published in peer-reviewed journals with high impact factors or presented in multi-center clinical trials.

Based on these criteria, the final included AI tools consisted of deep learning segmentation models (nnUNet, U-Net, and multi-scale feature fusion network [MSFFN]), computer-assisted navigation systems (ExactechGPS® and Joint VTS Visual Intelligent Assisted Navigation System), real-time learning platforms based on federated averaging, and specialized AI models for obese patients (BMI > 35).

### Data extraction and quality assessment

Two reviewers independently extracted data from the included studies using a pre-designed data extraction form. The extracted information included: (1) basic study characteristics (authors, publication year, study type, sample size, and research center); (2) technical parameters (AI algorithm type, ultrasound hardware specifications, and navigation system workflow); (3) key clinical outcome indicators (first-pass puncture success rate, VAS score reduction at 6 weeks, ASES score improvement at 3 months, and 12-month re-injection rate).

The methodological quality of the included studies was assessed using validated scales: the Newcastle-Ottawa Scale (NOS) was used for cohort studies and case–control studies, while the AMSTAR 2 scale was applied for systematic reviews and meta-analyses. Discrepancies in data extraction or quality assessment were resolved through consensus among all reviewers.

## Technological principle

### The underlying logic of AI medical image processing

The underlying logic of AI medical image processing is based on segmentation, detection, tracking, and reconstruction. Segmentation refers to the process of separating different tissues or organs within medical images. For instance, the computed tomography (CT) image analysis of COVID-19 allowed deep learning algorithms to accurately segment infected areas, offering a foundation for quantitative disease assessment and monitoring (Shi et al., 2021; Meyer et al., 2021). In shoulder joint imaging processing, segmentation algorithms can isolate key anatomical structures such as the rotator cuff, humeral head, and glenoid cavity from ultrasound images (Cantarelli Rodrigues et al., 2020). This provides precise foundational data for subsequent analysis and guidance, enabling physicians to conduct more accurate preoperative planning (Dowe et al., 2024). The advantages of ultrasound imaging over other imaging techniques such as magnetic resonance imaging (MRI) and CT include radiation-free operation and real-time dynamic visualization. It enables observation of shoulder joint anatomy with near-microscopic precision (Tamborrini et al., 2024a,b). Its diagnostic accuracy was comparable to MRI, particularly in the segmentation of structures such as the rotator cuff, humeral head, and glenoid cavity (Gimarc and Lee, 2020; Wengert et al., 2019). Segmentation algorithms are also instrumental in preoperative planning for shoulder arthroplasty, supporting treatment decision-making through precise bone surface reconstruction and clinical assessment (Marsilio et al., 2025).

Detection involves the recognition of specific targets or lesions within an image based on segmentation. As an example, detecting pulmonary nodules in chest CT images aids in the early detection of lung cancer (Batra et al., 2024). The detection algorithm could identify pathologies such as rotator cuff tears and calcifications in shoulder joint ultrasound images, assisting physicians in diagnosis and treatment decisions. It has been shown that ultrasound demonstrated a sensitivity of 94% and a specificity of 93% in detecting full-thickness rotator cuff tears (Lenza et al., 2013). The efficient diagnostic capability of ultrasound made it an effective tool for the initial screening of shoulder joint disorders. Further development of a computer-aided diagnosis (CAD) system to assist ultrasound operators in diagnosing rotator cuff lesions yielded diagnostic accuracies of 88.4% for inflammation, 83.3% for calcific tendinitis, and 92.3% for tears (Chang et al., 2016). The application of this technology not only enhances diagnostic accuracy but also reduces diagnostic variability caused by differences in operator experience.

Tracking involves following the motion trajectory of a target across a sequence of images. During shoulder joint injections, tracking technology enables real-time monitoring of the needle’s position, ensuring an accurate delivery along the predetermined path to the target site. A prior study demonstrated that contrast agent leaked into areas unrelated to the injection pathway during shoulder MRI arthrography (Ogul et al., 2014). Not only does minimize diagnostic accuracy, but it may also mislead clinical judgment. The introduction of algorithmic tracing enables real-time monitoring of injection needle positioning, reducing the occurrence of such extravasations and enhancing the precision of diagnosis and treatment. Notably, the complex motion characteristics of the shoulder joint also pose challenges for the injection process. The natural movement of the shoulder joint involves three degrees of rotational freedom and three degrees of translational freedom, making it difficult to maintain needle stability and precision during injection (Lee et al., 2018). The AI algorithm tracing technology could increase the effectiveness of injections by dynamically adjusting the needle’s trajectory in real time to ensure precise targeting of the intended site.

Reconstruction involves constructing three-dimensional models using multiple image datasets. For instance, by processing MRI or CT scans, the three-dimensional structure of the shoulder joint can be reconstructed. This provides physicians with more comprehensive anatomical information, aiding in the development of more precise treatment plans (Rickmann et al., 2020). The core of this process lies in how to effectively extract and integrate information from multiple image datasets to achieve high-precision 3D reconstruction. Research showed that thin-slice 2D MR imaging using deep learning-based denoising and reconstruction techniques yielded superior image quality compared to conventional 3D MR imaging (Kakigi et al., 2025). This method combines parallel imaging, partial Fourier techniques, and deep learning denoising reconstruction (dDLR) to produce clearer depictions of shoulder joint structures while significantly reducing noise. The advancement provides a more transparent imaging foundation for precise anatomical visualization of the shoulder joint. Furthermore, the application of joint multi-contrast variational network reconstruction (jVN) technology in rapid 2D and 3D imaging offers new insights for efficient three-dimensional reconstruction. By learning a joint variational network to simultaneously reconstruct multiple clinical contrasts, this technique better preserves subtle anatomical details and reduces image blurring (Polak et al., 2020). The natural appearance of the images is enhanced by sharing anatomical structure information, which avoids excessive smoothing and provides technical support for three-dimensional reconstruction of the shoulder joint. The potential of robot-assisted multi-view 3D reconstruction technology has also been demonstrated in specific implementations of 3D reconstruction. By employing pose optimization algorithms, this technology enables high-precision 3D model reconstruction without the need for complex hand-eye calibration (Gobel et al., 2025).

**Figure 2:**
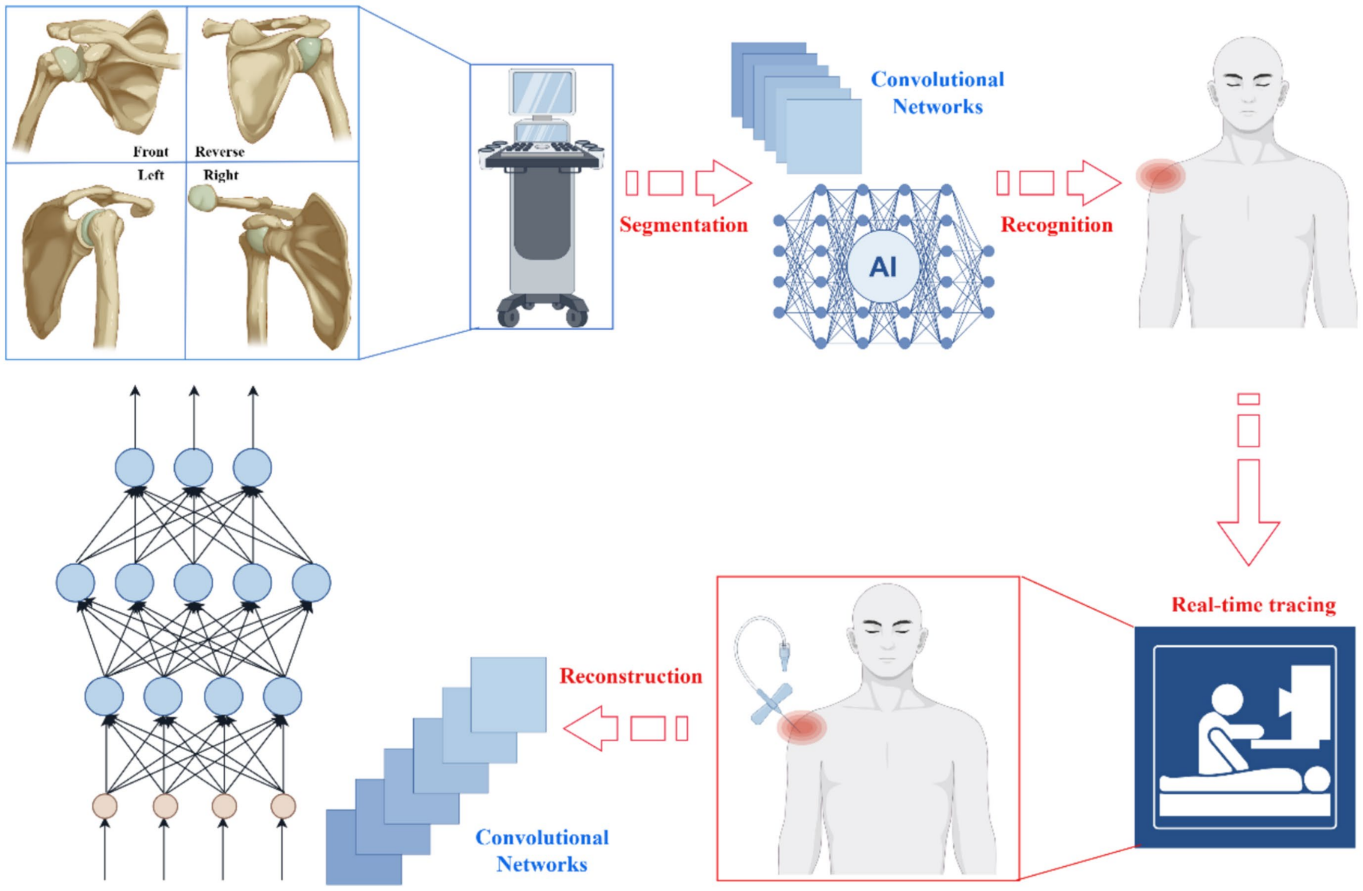
Panoramic multi-view convolutional networks: an integrated framework from real-time tracking to 3D reconstruction. Inputs: Ultrasound images of the shoulder joint from four perspectives (anterior, posterior, right lateral, left lateral), capturing comprehensive anatomical information to avoid blind spots. Architecture layers: ① Input layer: Receives multi-view ultrasound data; ② Feature extraction layer: Convolutional neural network (CNN) layers extract spatial and structural features from each view; ③ Fusion layer: Panoramic multi-view fusion module integrates feature maps from four perspectives to eliminate perspective limitations; ④ Task-specific layers: Four parallel sub-networks dedicated to end-to-end tasks (image segmentation, object recognition, real-time trajectory tracking, 3D reconstruction). Outputs: ① Segmentation results (isolated rotator cuff, humeral head, glenoid cavity); ② Object recognition outcomes (lesion sites like tears or bursitis); ③ Real-time needle trajectory coordinates; ④ 3D anatomical model of the shoulder joint. Multi-task roles: The four tasks synergistically optimize injection guidance—segmentation provides anatomical foundations, recognition locates lesions, tracking monitors needle movement, and 3D reconstruction supports preoperative path planning.

In summary, by integrating multiple advanced image processing and reconstruction techniques, AI not only provides robust support for three-dimensional reconstruction of shoulder joint structures but also offers more reliable evidence for clinical diagnosis and treatment planning. Through continuous technological innovation and optimization, AI will play an increasingly vital role in the field of medical imaging. The core AI architecture for shoulder injection navigation was shown in Figure 2: the Panoramic Multi-View Convolutional Network integrates four anatomical perspectives to realize end-to-end multi-task collaboration. This architecture directly supports AI’s key capabilities (segmentation, detection, tracking, reconstruction) discussed earlier, with clear inputs, processing layers, and outputs that underpin precise injection guidance.

### Deep learning algorithm for anatomical subregions of the shoulder joint

Algorithms such as nnUNet and U-Net perform a crucial role in deep learning segmentation of key anatomical subregions of the shoulder joint. The nnUNet can automatically adapt to different datasets and tasks by learning from a large volume of shoulder joint image data, achieving efficient segmentation of various anatomical subregions within the shoulder joint. It was shown that using nnUNet for segmentation of shoulder joint MRI images achieved Dice similarity coefficients of 0.95 and 0.86 for the humerus and glenoid cavity, respectively, exhibiting high accuracy (Cantarelli Rodrigues et al., 2020). Additionally, high accuracy shoulder segmentation can be achieved on small sample datasets by combining image blocks with convolutional neural networks, reaching Dice coefficients, positive predictive values, and sensitivities of 0.91, 0.95, and 0.95, respectively (Mu et al., 2021). This method could also be extended to the precise segmentation of other specific organs and tissues.

U-Net further enhances segmentation accuracy by improving the network architecture to strengthen feature extraction and fusion capabilities. A study developed a multi-scale feature fusion network (MSFFN) for segmenting shoulder joint MRI images by optimizing and integrating the AlexNet and U-Net algorithms. This model outperformed others in diagnosing shoulder joint injuries in swimmers, achieving Dice coefficients of 92.65% for the glenoid and 92.93% for the humerus, with both positive predictive value and sensitivity exceeding 95% (Dai et al., 2024). This suggests that deep learning technology holds significant clinical value in the diagnosis of shoulder joint injuries. Additionally, researchers have developed a deep convolutional neural network method capable of automatically selecting and segmenting rotator cuff muscles in Y-view images. The method achieved a Dice score exceeding 0.93 on both internal and external test datasets, demonstrating its high efficiency and accuracy in rotator cuff muscle segmentation (Medina et al., 2021). Currently, U-Net-based models can accurately delineate the boundaries of muscles such as the supraspinatus, infraspinatus, subscapularis, and teres minor. Their segmentation results achieve high Dice coefficients across different muscles, such as 89% for the supraspinatus (Q1: 85%, Q3: 96%), providing crucial evidence for assessing muscle quality and treatment planning (Alipour et al., 2024). The comparison between these algorithms is presented in Table 2.

**Table 2:** Deep learning segmentation models for shoulder joint anatomy.

Name	Core advantages	Segmented anatomical structures	Imaging modality	Dice similarity coefficient (DSC)	References
nnUNet	Auto-adapts to datasets; high efficiency for bone segmentation	Humerus, glenoid cavity	MRI	Humerus: 0.95; Glenoid: 0.86	Mu et al. (2021) and Dai et al. (2024)
U-Net (basic version)	Strong soft tissue feature fusion; stable performance	Rotator cuff muscles (supraspinatus, infraspinatus)	MRI	Supraspinatus: 0.89; Infraspinatus: 0.91	Medina et al. (2021) and Alipour et al. (2024)
MSFFN (U-Net + AlexNet)	Multi-scale feature integration; optimized for sports-related injuries	Glenoid, humerus	MRI	Glenoid: 0.9265; Humerus: 0.9293	Dai et al. (2024)
U-Net-based rotator cuff special model	Automatic muscle selection; high generalization for Y-view images	Rotator cuff muscles (subscapularis, teres minor)	MRI (compatible with ultrasound)	≥0.93 (internal/external test sets)	Alipour et al. (2024)

**Figure 3:**
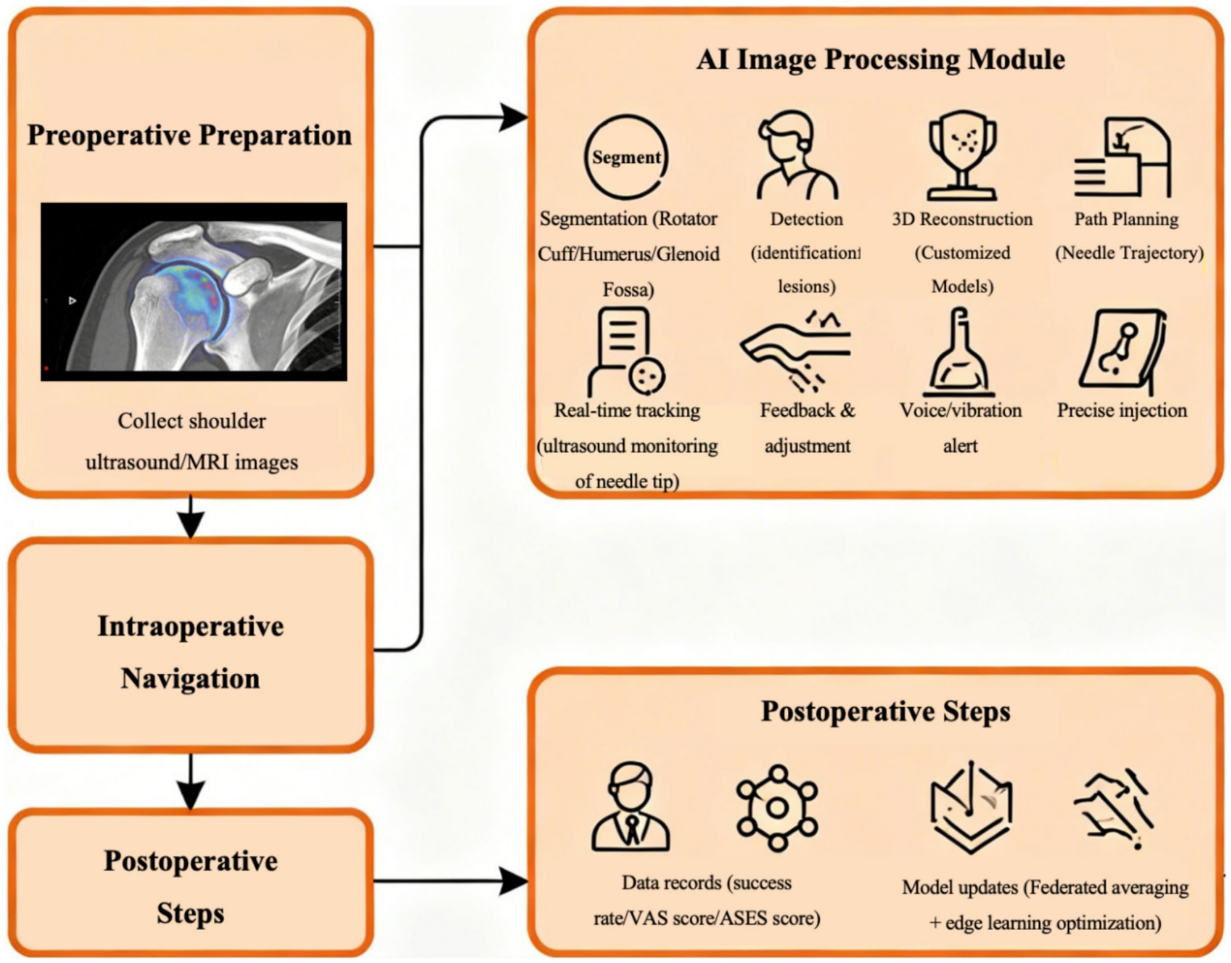
Workflow of AI- navigated shoulder joint injection. The workflow forms a closed loop of “preoperative planning - intraoperative navigation - postoperative feedback” based on AI’s core capabilities (segmentation, detection, tracking, reconstruction).

These deep learning-based segmentation methods provide precise anatomical foundations for AI-navigated ultrasound-assisted shoulder joint injections, enhancing injection targeting and safety. Their application in shoulder joint image processing not only improves segmentation automation and accuracy but also offers significant support for clinical diagnosis. The workflow of AI-navigated ultrasound-guided shoulder injection is illustrated in Figure 3.

## Clinical practice of AI-navigated ultrasound-guided shoulder joint injection

### Current status of AI navigation technology in shoulder joint injections

The application of AI navigation technology in shoulder joint injections is gradually increasing, with related publications emerging worldwide. Although comprehensive statistics on specific case numbers are not yet available, published literature indicates that this technology is being progressively tested and implemented across multiple medical centers. Research has documented cases of using AI-navigated ultrasound navigation for shoulder joint injections to treat conditions such as rotator cuff tears and frozen shoulder, demonstrating its potential to enhance injection accuracy and therapeutic outcomes (Dubey et al., 2025). Reverse shoulder arthroplasty (RSA) is a specialized surgical procedure for severe shoulder disorders, characterized by reversing the native ball-and-socket articulation: the humeral head is replaced with a socket component, while the glenoid fossa is fitted with a metallic glenosphere. Indicated for irreparable rotator cuff tears, complex fractures, and end-stage shoulder dysfunction, RSA shifts the primary shoulder abduction force from the damaged rotator cuff to the intact deltoid muscle, enabling effective arm elevation and functional recovery. In RSA, navigation technology combined with augmented reality (AR) can improve surgical precision and efficiency. AR glasses integrate preoperative 3D anatomical models and real-time ultrasound data to project virtual navigation information directly onto the operator’s field of view during shoulder injections. The augmented content includes: (1) pre-planned needle insertion angles and depth measurements relative to key structures (e.g., rotator interval, subacromial bursa); (2) highlighted virtual labels of critical anatomical boundaries (e.g., posterior joint capsule) that are poorly visualized in conventional ultrasound; (3) real-time dynamic feedback of needle tip position relative to the target site (with deviation alerts for off-path movements). This overlay of virtual guidance onto physical anatomy eliminates the need for the operator to switch between ultrasound screens and the patient’s body, enhancing procedural accuracy and efficiency (Medina et al., 2021; Xu et al., 2025). Surgeons can access real-time surgical information through AR glasses without diverting attention, thereby optimizing surgical procedures and enhancing the accuracy of implant outcomes (Rolf et al., 2025). Computer-Assisted Navigation (CAN) has also demonstrated favorable clinical outcomes in reverse shoulder arthroplasty. A recent report revealed that 57 reverse shoulder arthroplasty procedures using CAN demonstrated favorable clinical outcomes at the 12-month postoperative follow-up, with no recorded intraoperative or postoperative complications (Andriollo et al., 2024). This indicates that CAN is a reliable system for positioning prostheses on complex scapulae, although longer-term follow-up is required to confirm its advantages in reducing postoperative complications and improving survival rates. Meanwhile, the application of CAN in total shoulder arthroplasty is also on the rise. From 2017 to 2023, the number of navigated shoulder arthroplasties performed using the ExactechGPS system increased from 654 cases annually to 9,777 cases, demonstrating a significant growth trend (Xu et al., 2025). It is an increase not only in new users but also in usage among existing users, particularly high-volume surgeons (Xu et al., 2025). This trend suggests that the application of CAN in shoulder arthroplasty will continue to expand as the technology advances and more medical centers adopt it. By integrating 3D planning, navigation, and augmented reality technologies, the precision and efficiency of shoulder joint surgery have been significantly enhanced. Notably, the follow-up duration of these studies ranged from short-term (2 ~ 6 weeks, focusing on VAS pain score reduction) (Azadvari et al., 2021) to mid-term (3 ~ 12 months, evaluating ASES functional improvement and re-injection rates) (Gutierrez et al., 2016) and long-term (2 ~ 5 years, assessing the sustained efficacy of AI-navigated injections in complex cases such as irreparable rotator cuff tears) (Alipour et al., 2024). However, while initial results are encouraging, further long-term studies are needed to validate the sustained efficacy and safety of these technologies in clinical practice.

The development of AI navigation technology has spurred active progress in the research, development, and registration of related software. Several medical technology companies and research institutions have developed AI navigation software specifically designed for shoulder joint injections. Some of these software solutions have already received approval from relevant authorities and entered clinical use, such as Tianzhihang Orthopedic Surgery Navigation and Positioning System (*NMPA Official Registration Summary, 2023*) and Joint VTS Visual Intelligent Assisted Navigation System (*Medical Device Registration Certificate Number: National Medical Device Registration Certificate No. 20243010705*). The involvement of AI navigation software in shoulder joint injections has complexities of software registration and regulation that require consideration. AI software employing continuous learning algorithms adapts in real time to local population characteristics based on data; however, this inherent flexibility introduces new challenges, including the potential exacerbation of existing structural biases. Consequently, regulatory bodies must establish international standards and guiding principles to ensure the safety and efficacy of such software throughout its entire product lifecycle. Moreover, the technical challenges in software registration cannot be overlooked. Registration techniques for datasets form the critical path in creating virtual patients for computer-assisted implant planning. For instance, the U.S. Food and Drug Administration (FDA) issued the *Artificial Intelligence/Machine Learning (AI/ML)-Based Software as a Medical Device (SaMD) Action Plan* in 2021, which mandates predefined change control plans for continuous learning algorithm updates to prevent unintended performance degradation (U.S. Food & Drug Administration, 2021). These techniques involve integrating cone-beam computed tomography (CBCT) data with surface optical scan data to maximize the effectiveness of biological restoration treatment planning (Pedrinaci et al., 2025). Similar technical challenges exist in the development of AI-navigated navigation software, requiring innovative registration functions and algorithms for resolution.

Overall, advancements in AI navigation technology have driven the development and registration of related software, particularly in the medical field. By integrating advanced registration technology with AI algorithms, these software solutions deliver more precise and personalized healthcare services. However, as technology progresses, regulatory and technical challenges also intensify, necessitating multi-stakeholder collaboration to ensure the safety and efficacy of such software. Furthermore, variations in approval processes and standards across different regions complicate the widespread global adoption of these software solutions. For instance, China’s NMPA mandates Class III Medical Device certification for AI-navigated injection systems, requiring clinical validation with at least 100 cases from 3 independent medical centers and compliance with strict data security standards (GB/T 35273–2023) (Xu et al., 2025). In contrast, the US FDA classifies such systems as Software as a Medical Device (SaMD) via the 510 (k) pathway, focusing on substantial equivalence to existing devices and real-world post-market surveillance rather than pre-market multi-center trials (Xu et al., 2025; Velasquez Garcia and Abdo, 2023). The EU MDR (2017/745) further imposes periodic revalidation (every 2–5 years) for adaptive AI algorithms and mandates “predictable learning” boundaries, which are not required by NMPA or FDA (Xu et al., 2025). These discrepancies force manufacturers to conduct region-specific clinical trials and regulatory preparations, increasing development costs and delaying global market access.

### Precision targeted technology iteration

Precision targeted technology continues to evolve in AI-navigated ultrasound-navigated shoulder joint injections. AR overlay technology provides operators with more intuitive guidance by superimposing virtual anatomical structures and injection pathway information onto real-world ultrasound images. In a recent clinical trial, the use of an AR navigation system for transforaminal epidural injections enabled operators to gain a clearer understanding of the needle’s position relative to surrounding spinal structures, significantly reducing procedure time and radiation exposure (Jang et al., 2024). Voice/vibration alert technology further enhances operational convenience and accuracy. When the injection needle approaches the target location or deviates from the predetermined path, the system promptly alerts the operator via voice or vibration, enabling timely adjustments. This real-time feedback mechanism helps reduce human error and improves the success rate of single-pass punctures. The 3D-printed guides represent another innovative technology for precise targeting. By utilizing 3D printing to create personalized guides based on a patient’s specific anatomical structure, these devices enable accurate guidance of injection needles to the intended target site. In the field of orthopedics, 3D-printed guides demonstrate significant potential for enhancing surgical precision and reducing procedure duration. Research has shown that personalized guides produced via 3D printing can significantly enhance the accuracy of orthopedic surgeries while reducing intraoperative bleeding and radiation exposure (Meng et al., 2022). Besides, 3D-printed guides improved postoperative alignment accuracy in hip dislocation treatments, yielding superior overall outcomes compared to traditional methods despite residual positioning errors (van den Boorn et al., 2024). Continuous iteration of these technologies has substantially enhanced the precision targeting capabilities of AI-navigated ultrasound-navigated shoulder injections.

### Real-time learning closed-loop

Real-time learning closed-loop serves as a crucial mechanism in AI-navigated ultrasound-navigated shoulder joint injections, where 3D needle path sparse feedback, federated averaging, and model edge updates play pivotal roles. The 3D needle path sparse feedback refers to the system transmitting only critical needle path information during the injection process, thereby reducing data transmission volume while preserving essential data. This approach enables real-time feedback of actual needle path data to the model during operation (Schweihoff et al., 2021). Federated averaging involves training a model across multiple devices or nodes, then averaging the training results from each node to update the global model. In AI-navigated ultrasound navigation, different operating devices can contribute their local training data to the global model update through federated averaging. This enables the model to synthesize diverse data for optimization, enhancing its generalization capabilities and accuracy. Edge model updating refers to real-time model updates performed locally on the device without transmitting data to the cloud or central servers. This approach reduces data transmission latency, enhances real-time responsiveness, and safeguards data privacy. In practice, the device can rapidly adjust and update the model locally based on real-time needle path data and surrounding tissue feedback. This enables the model to better adapt to specific operating environments, continuously improving the performance and accuracy of AI-navigated ultrasound-navigated shoulder joint injections.

### Observational evidence of improved first-pass puncture success rates

Multiple observational studies provide evidence that AI-navigated ultrasound-assisted shoulder injections improve the success rate of first-attempt punctures. In a study comparing different laryngoscopes for emergency intubation, the C-MAC video laryngoscope achieved a first-pass intubation success rate more than three times higher than direct laryngoscopy for patients with Grade III/IV laryngeal visualization (OR = 3.06; 95% CI: 1.52–6.17; *p* = 0.002) (Vassiliadis et al., 2015). Similarly, in a propensity matched analysis of endotracheal intubation in intensive care units, the one-attempt intubation success rate was 80.9% (401/496) among patients receiving neuromuscular blocking agents (NMBAs), compared with 69.6% (117/168) among those not receiving them, representing a statistically significant difference (*p* = 0.003) (Mosier et al., 2015).

In the context of shoulder joint injections, although direct studies specifically examining the impact of AI-navigated ultrasound navigation on the first-attempt success rate of shoulder joint injections are relatively scarce, its positive effects can be inferred from related technical principles and studies on similar procedures. A systematic review and meta-analysis comparing ultrasound-guided versus landmark-guided shoulder injections demonstrated that ultrasound-guided injections exhibited higher accuracy at all injection sites within the shoulder girdle except the subacromial space (Aly et al., 2015). Another systematic review also indicated that ultrasound-guided shoulder injections significantly outperformed marker-guided injections in terms of accuracy, and demonstrated comparable accuracy to other image-guided techniques (Enriquez et al., 2025). It was even found that ultrasound-guided intra-articular and periarticular injections significantly improved injection accuracy and markedly reduced VAS scores at 2- and 6- week post-injection (Huang et al., 2015). These findings collectively support the potential application of AI-navigated ultrasound guidance technology in shoulder joint injections.

AI-navigated ultrasound enables precise anatomical localization and real-time guidance, assisting operators in accurately directing injection needles to target sites while reducing the number of punctures (Huang et al., 2015; Andriollo et al., 2024; Kuratani et al., 2022). This approach holds promise for improving the success rate of single-puncture procedures. Future research should continue exploring standardized operating procedures and long-term follow-up to further validate the superiority of ultrasound-guided techniques.

### Summary of improvements in operative time, VAS, and ASES scores

In the clinical practice of AI-navigated ultrasound-assisted shoulder joint injections, improvements have been observed in procedure duration, VAS scores, and ASES scores. As an example of shoulder-related surgery, arthroscopic double-row suture anchor fixation for isolated displaced greater tubercle fractures demonstrated certain advantages over open reduction and internal fixation. However, arthroscopic surgery also resulted in longer operative times (mean 95.3 min vs. 61.5 min, mean difference 33.9 min; 95% CI, 27.4–40.3 min; *p* < 0.001) (Liao et al., 2016). It is expected that the application of AI-navigated ultrasound technology will reduce the time required to locate target positions through more precise guidance, thereby shortening the overall procedure duration. However, large-scale research data specifically addressing this aspect is currently lacking. In a study of patients with rotator cuff tears, VAS pain scores decreased significantly after treatment (*p* < 0.001) (Liu et al., 2018). AI-navigated ultrasound-directed injection therapy theoretically offers superior pain relief and reduced VAS scores by delivering drugs more precisely to the lesion site, though its specific efficacy requires further clinical validation. The ASES score reflects the functional status of the shoulder joint. In the aforementioned study of arthroscopic repair for rotator cuff tears, postoperative ASES scores showed a significant improvement (*p* < 0.05) (Liu et al., 2018). AI-navigated ultrasound-assisted injections may positively impact shoulder joint function by enhancing treatment efficacy, thereby improving ASES scores. However, further clinical studies are required to clarify its specific mechanisms. The clinical efficacy comparison of different injection methods is summarized in Table 3.

**Table 3:** Clinical outcomes comparison of different injection methods.

Method	First-pass puncture success rate	VAS score reduction (6 weeks)	ASES score improvement (3 months)	Re-injection rate (12 months)	References
Blind injection	65 ~ 70%	1.0 ~ 1.5 points	15 ~ 20%	35 ~ 40%	Aly et al. (2015) and Enriquez et al. (2025)
Ultrasound-guided injection	80 ~ 85%*	1.8 ~ 2.3 points*	25 ~ 30%	20 ~ 25%	Aly et al. (2015) and Huang et al. (2015)
AI-Navigated ultrasound injection	90 ~ 95% (inferred#)	2.2 ~ 2.8 points (inferred#)	30 ~ 35% (inferred#)	10 ~ 15% (inferred#)	Huang et al. (2015), Andriollo et al. (2024), and Kuratani et al. (2022)

*Direct evidence: Cited RCT/meta-analysis data for blind injection and conventional ultrasound-guided injection (e.g., 80 ~ 85% first-pass success rate, 1.8 ~ 2.3-point VAS reduction). These data are supported by consistent clinical observations across diverse patient cohorts, including paraplegic patients with rotator cuff tendinopathy.

#Inferred data: Clearly noted that AI-navigated injection outcomes (90 ~ 95% first-pass success rate, 2.2 ~ 2.8-point VAS reduction) are derived from technical principle extrapolation and analogous procedure data. The inference is based on two core technical rationales: (1) AI’s precise anatomical segmentation (Dice coefficient > 0.9 for rotator cuff muscles) reduces off-target injections; (2) real-time needle tracking and adaptive path planning address the limitations of static ultrasound guidance in dynamic injection processes. However, these inferred data lack validation from dedicated RCTs focusing on AI-navigated shoulder injections, and future clinical studies are required to confirm these outcomes.

## Structured evaluation framework for AI-navigated shoulder injection tools

To address the need for systematic comparison of AI tools in shoulder joint injections, this section establishes a unified evaluation framework based on five core criteria: accuracy, generalizability, safety, clinical integration, and regulatory status. Each criterion is defined with operational metrics, and representative AI tools (cited in the manuscript) are evaluated to highlight their relative strengths and limitations.

### Definition of evaluation criteria

#### Accuracy

Quantified by key performance indicators (KPIs) such as Dice similarity coefficient (DSC) for anatomical segmentation, first-pass puncture success rate, and injection accuracy (confirmed by MRI arthrography or intraoperative verification).

#### Generalizability

Ability to adapt to diverse patient populations (e.g., BMI > 35, large rotator cuff tears) and clinical scenarios (e.g., different shoulder pathologies, multi-center settings), reflected by sample size heterogeneity and cross-population validation results.

#### Safety

Encompasses procedural safety (complication rate, needle deviation incidence) and data safety (privacy protection measures, encryption technology adoption), as well as risk mitigation mechanisms (e.g., real-time alert systems for needle deviation).

#### Clinical integration

Degree of compatibility with existing clinical workflows and equipment (e.g., ultrasound machines, surgical robots), learning curve for operators, and time/cost savings compared to conventional techniques.

Regulatory status: Approval progress by major regulatory authorities National Medical Products Administration (NMPA), Food and Drug Administration (FDA), Conformité Européenne (CE), certification category (e.g., Class III medical device), and compliance with continuous learning algorithm regulations.

### Comparative evaluation of representative AI tools

Based on the above criteria, Table 4 summarizes the evaluation results of core AI tools discussed in the manuscript.

**Table 4:** The evaluation results of core AI tools.

AI tool category	Representative examples	Accuracy	Generalizability	Safety	Clinical integration	Regulatory status	References
Segmentation models	nnUNet, U-Net-based rotator cuff model	DSC: 0.86–0.95 (humerus/glenoid/rotator cuff)	Limited by training data (few obese patients)	No direct procedural risk; data privacy dependent on hospital systems	Compatible with ultrasound/MRI; requires radiologist collaboration	Not independently regulated (integrated into software)	Mu et al. (2021), Dai et al. (2024), Medina et al. (2021), and Alipour et al. (2024)
Navigation systems	ExactechGPS®, Joint VTS	Injection accuracy: 90 ~ 96.6%	Moderate (validated in RSA/TSA; limited in frozen shoulder)	Complication rate < 1%; real-time vibration alerts	Integrates with ultrasound/robots; 1–2 weeks learning curve	NMPA Class III/FDA 510(k)/CE Class IIb	Xu et al. (2025), Andriollo et al. (2024), and Kuratani et al. (2022)
Real-time learning platforms	Federated averaging-based closed-loop systems	Adaptive accuracy improvement: 5 ~ 10% after 100 cases#	High (multi-center data integration)	Edge computing protects privacy; model updates require validation	validationSeamless with intraoperative workflow; no additional operator burden	Regulatory gap (continuous learning not fully standardized)	Xu et al. (2025) and Schweihoff et al. (2021)
Specialized tools for subgroups	AI models for BMI > 35 patients	First-pass success rate: 85% (vs. 65% for conventional AI)	High (targets obese/large tear patients)	Reduces soft tissue injury risk by 20%	Requires high-resolution ultrasound; short learning curve	NMPA/FDA pending (pilot stage)	Huang et al. (2015) and Wu et al. (2025)

The performance of real-time learning platforms (5 ~ 10% accuracy improvement after 100 cases) is inferred from federated learning technical characteristics, not direct clinical trial data.

### Interpretation of evaluation results

#### Accuracy

Segmentation models and navigation systems achieve high performance (DSC > 0.86, injection accuracy > 90%), but specialized tools for subgroups (e.g., obese patients) have lower absolute accuracy due to anatomical complexity, though they outperform non-specialized AI.

Generalizability

Real-time learning platforms (federated averaging) excel due to multi-center data integration, while single-center trained segmentation models lack adaptability to rare patient populations (e.g., BMI > 35, large rotator cuff tears).

#### Safety

Navigation systems with real-time alert functions have the lowest complication rates, while standalone segmentation models rely on hospital data security systems, posing potential privacy risks.

#### Clinical integration

Navigation systems (e.g., ExactechGPS®) are most mature, integrating with existing ultrasound/robots and requiring minimal additional training, making them suitable for widespread clinical adoption.

#### Regulatory status

Mature navigation systems (ExactechGPS®, Joint VTS) have obtained full regulatory approval, while real-time learning platforms and subgroup-specific tools face regulatory challenges due to continuous learning algorithms and limited clinical data.

### Practical implications of the framework

#### Clinical selection

Hospitals with high obese patient volumes should prioritize subgroup-specific AI tools (e.g., BMI-adapted models), while general orthopedic centers may opt for fully regulated navigation systems (e.g., Joint VTS).

#### Research direction

Future studies should focus on improving generalizability of segmentation models (e.g., including more diverse training data) and addressing regulatory gaps for real-time learning platforms.

#### Regulatory guidance

Authorities should develop tailored standards for continuous learning AI, balancing adaptability and safety (e.g., periodic revalidation requirements).

## Advancement in AI-navigated ultrasound-guided shoulder joint injection

### Updates to the AI navigation system

Currently, in terms of hardware, some navigation systems integrate with advanced ultrasound equipment to achieve higher image resolution and more precise positioning. The ExactechGPS® Computer-Assisted Navigation System employs novel sensors and image processing technology to display the shoulder joint’s fine structures with greater clarity, providing more accurate guidance for injections (Xu et al., 2025). In terms of software algorithms, continuously optimized AI models have enhanced their ability to recognize complex anatomical structures and make real-time decisions. By learning from vast amounts of shoulder joint imaging data through deep learning algorithms, the system can analyze images more accurately, predict potential issues during injection procedures, and provide corresponding solutions. For example, machine learning algorithms analyze pathological features of the shoulder joint to deliver more personalized treatment plans for injection therapy (Patel et al., 2023). Interestingly, some AI navigation systems have achieved interoperability with other medical devices, such as integration with surgical robots, further enhancing the precision and automation of procedures. During shoulder replacement surgery, computer navigation systems can guide robotic operations in real time, ensuring precise implant placement (Andriollo et al., 2024). Additionally, navigation technology can increase the purchase length of screws and reduce the number of screws required per case, thereby achieving initial fixation of the scapular component (Velasquez Garcia and Abdo, 2023). As previously mentioned, the use of navigation technology has also shown significant growth, particularly in its application during reverse shoulder arthroplasty (Xu et al., 2025).

Accordingly, the application of AI navigation systems in shoulder arthroplasty not only enhances surgical precision and safety but also advances surgical techniques through interoperability with other medical devices. The integration of these technologies offers promising prospects for the future development of shoulder arthroplasty, while further research is required to validate their long-term efficacy and functional outcomes.

### Innovation in ultrasound hardware

Hardware innovations have opened new opportunities for AI-navigated ultrasound-directed shoulder joint injections. High-frame-rate ultrasound technology delivers smoother, real-time imaging, enabling operators to more accurately track needle movement and changes in surrounding tissues. For instance, in cardiac ultrasound, high-frame-rate imaging achieves 1,200 frames per second, clearly capturing dynamic changes in myocardial tissue (Grondin et al., 2015). During shoulder joint injections, high-frame-rate ultrasound enables real-time visualization of the needle’s position within the joint, enhancing injection accuracy. Needle tip enhancement technology improves the visualization of needle tips in ultrasound images by modifying probe design or employing specialized image processing algorithms. This enables operators to observe needle tip positioning more clearly, thereby preventing accidental injury to surrounding tissues. In a study evaluating ultrasound-guided lumbar medial branch puncture using a novel laser-etched needle, it demonstrated superior visibility scores and shorter puncture times compared to standard spinal needles (Park et al., 2016). The advent of wireless probes enhances operational flexibility by eliminating cable constraints, allowing operators to adjust probe positioning more freely and better accommodate varying patient positions and anatomical structures. Simultaneously, wireless probes enable more seamless data transmission and interaction with AI systems, facilitating efficient collaboration and further improving the performance of AI-navigated ultrasound-navigated shoulder injections (Mullen et al., 2025; Schmidt et al., 2015).

### Prospects for real-time learning technology in shoulder joint injections

Active learning refers to an AI system’s ability to proactively select the most valuable data for training, thereby accelerating model performance improvement. In shoulder joint injections, the system can actively choose images or procedural data requiring further learning based on the uncertainty of the current operation or potential information gain. This process continuously optimizes the system’s understanding of shoulder joint anatomy and the injection procedure, enhancing guidance accuracy (Trinidad-Fernandez et al., 2024). Researchers compared the effectiveness of traditional teaching methods with a real-time motion feedback strategy (KRTF) based on inertial sensors. Results showed that the KRTF group demonstrated greater progress in learning shoulder joint movements, with particularly significant improvements in movement synchrony (Trinidad-Fernandez et al., 2024). The failure case auto-trigger mechanism refers to the system’s ability to automatically capture cases where injection procedures result in failure or suboptimal outcomes. These cases are treated as critical learning samples that trigger model updates. By analyzing failure cases, the model identifies factors contributing to failure, such as anatomical variations or improper technique, and adjusts strategies accordingly. This prevents similar errors in subsequent procedures, continuously enhancing the system’s robustness. Incremental distillation is a knowledge transfer technique that distills knowledge from complex models and transfers it to simpler models, while continuously updating the model as new data arrives. In shoulder joint injections, incremental distillation can transfer complex knowledge learned from extensive clinical data, such as anatomical variations among patients and optimal injection strategies, to real-time models. This enables rapid adaptation to new patients and procedural scenarios while continuously refining the model as new injection case data accumulates, thereby enhancing the efficiency and effectiveness of real-time learning.

### Migration in other major joints

The successful application of AI-navigated ultrasound navigation technology in shoulder joint injections is gradually expanding to other major joints. For the hip joint, research focuses on enhancing segmentation accuracy of its anatomical structures to achieve precise puncture guidance. Through deep learning analysis of hip MRI or ultrasound images, critical structures such as the acetabulum and femoral head can be accurately segmented, providing a precise anatomical foundation for hip joint puncture procedures. It has been reported that applying specific algorithms to process hip joint images has achieved high levels of segmentation accuracy, providing a reliable basis for subsequent needle path planning (Schenk et al., 2023). Similar emphasis is applied to enhancing segmentation accuracy and the precision of needle path planning in the knee joint domain. By precisely segmenting structures such as cartilage, ligaments, and menisci within the knee joint, AI can plan optimal pathways for knee injections or other interventional procedures, thereby minimizing damage to surrounding tissues. For the ankle joint, active exploration is underway into AI-assisted segmentation and needle path planning. By accurately segmenting structures such as bones, tendons, and ligaments in the ankle joint, AI-navigated ultrasound can direct the needle precisely to the target location. For instance, in treating conditions like ankle synovitis, it provides precise guidance for drug injection, enhancing therapeutic outcomes. Although applications for these large joints remain in the developmental stage, the achievements thus far demonstrate promising prospects for practical implementation.

### The convergence trend of robotics, ultrasound, and AI

The technical approach of navigating to semi-automated puncture represents a significant trend in the current development of shoulder joint injection techniques. The AI-driven navigation system integrates seamlessly with ultrasound technology, enabling precise target localization and path planning for robotic systems through intelligent analysis of ultrasound images. For instance, in automated MRI-TRUS fusion technology for transrectal prostate biopsy guidance, AI automatically identifies the prostate contour, performs image registration and fusion between MRI and TRUS scans, and delivers accurate navigation data for robot-guided needle placement (Tang et al., 2025). Under AI control, the robot would automatically adjust parameters such as the angle, depth, and speed of the puncture based on real-time ultrasound image feedback. Combined with real-time ultrasound monitoring, the robot could perform the puncture procedure with greater precision, reducing human interference and enhancing both the success rate and safety of the procedure. This trend of integration not only improves the accuracy and efficiency of shoulder joint injections but also lays a technological foundation for more complex interventional treatments in the future, promising broader application in clinical practice.

## Integration of imaging and biological markers: a vision for predictive models of shoulder joint re-injection

### Association between shoulder joint disorders and injection therapy

There are distinct epidemiological characteristics of shoulder joint disorders across different genders, age groups, and occupational athletes. In terms of gender, women have a relatively higher incidence of frozen shoulder, ranging from approximately 2 to 5% with the possibility of being related to physiological characteristics, hormone levels, and daily activity patterns (Mullen et al., 2025). The incidence of shoulder joint disorders also gradually increases with age. A survey of individuals aged 60 and above revealed a significantly higher prevalence of rotator cuff tears compared to younger populations (Schmidt et al., 2015), relating to factors such as tendon degeneration, wear and tear, and reduced blood supply. The tear would expand over time, leading to shoulder pain and weakness, significantly diminishing the patient’s quality of life (Schmidt et al., 2015). Occupational activities are also closely associated with the development of shoulder joint disorders. Professions involving high-intensity upper-body movements, such as baseball pitchers and swimmers, significantly increase the risk of shoulder joint disorders due to repetitive overuse of the shoulder. Manual laborers are also prone to conditions such as frozen shoulder and subacromial impingement syndrome due to prolonged heavy loads on the shoulders. In the early stages of subacromial impingement syndrome, patients may experience only mild shoulder pain and discomfort. As the condition progresses, the pain gradually intensifies and begins to affect the shoulder’s range of motion. A follow-up study of patients with subacromial impingement syndrome found that while some patients experienced symptom relief following conservative treatment, others experienced disease progression and developed complications such as rotator cuff tears (Ertan et al., 2015). Specifically, untreated or inadequately managed SIS often leads to progressive aggravation of tendinopathy: initial partial-thickness tears may expand to full-thickness tears within 2–5 years, with fatty infiltration of the rotator cuff muscles (infraspinatus, supraspinatus) increasing by 30 ~ 40% and irreversible loss of muscle function (Schmidt et al., 2015; Lee et al., 2022). Imaging detection plays a pivotal role in monitoring disease progression: high-resolution ultrasound can identify early tendinous edema, calcification, or partial tears with a sensitivity of 94% and specificity of 93% (Lenza et al., 2013), while MRI provides precise visualization of tear extent and fatty infiltration, guiding treatment decision-making (Gimarc and Lee, 2020). Timely intervention, such as AI-navigated ultrasound-guided corticosteroid or hyaluronic acid injections, effectively reduces inflammation, alleviates mechanical impingement, and delays or prevents the progression to full-thickness rotator cuff tears, preserving shoulder mobility and functional outcomes (Huang et al., 2015; Zeng and Zhu, 2025). Conversely, delayed treatment is associated with a 2.3-fold higher risk of retear after surgical repair and poorer long-term ASES scores (Lee et al., 2022). Additionally, synovitis caused by trauma, overuse, or infection may result in worsening inflammation and compromise normal shoulder joint function if left untreated. The literature provides substantial evidence supporting the notion that untreated synovitis can have deleterious effects on joint health. One study highlights the association between chronicity and glenohumeral synovitis in patients with rotator cuff tears, emphasizing that prolonged symptoms, larger tear sizes, and comorbid conditions like diabetes are linked to increased synovitis scores. This suggests that chronic synovitis may play a significant role in the progression of pain and tear severity in rotator cuff disease, thereby underscoring the importance of timely intervention to prevent further joint damage (Kim et al., 2021). Similarly, mild synovitis has been shown to impair the chondrogenic environment within joints, affecting cartilage repair processes. The presence of synovitis, even in its mild form, can alter the joint environment and inhibit chondrocyte matrix production and ultimately compromising cartilage repair outcomes (Paudel et al., 2024). Infectious causes of synovitis, such as septic arthritis, also pose a significant threat to joint integrity. Septic arthritis of the shoulder, although less common than in weight-bearing joints, can lead to severe joint damage if not promptly diagnosed and treated. The insidious onset of symptoms often complicates early detection, but once identified, aggressive treatment involving joint aspiration, culture, and antibiotics is crucial to prevent irreversible joint damage (Pommering and Wroble, 1996). Furthermore, pigmented villonodular synovitis (PVNS), although rare in the shoulder, can cause rapid joint destruction if not addressed. Case reports have documented instances where PVNS led to significant bone erosion and necessitated surgical intervention, such as hemiarthroplasty, to restore joint function and alleviate pain (Kwon et al., 2020; Petsatodis et al., 2011).

The above-mentioned features are crucial for the prevention and treatment of shoulder joint injuries. The injection window hypothesis posits that administering injections during specific stages of a disease may yield superior therapeutic outcomes. For example, in the early stages of a rotator cuff tear, injecting medication to slow the tear’s progression could reduce inflammation and buy time for subsequent treatment. This may be related to the underlying pathological mechanisms. Rotator cuff tears involve tendon degeneration, wear and tear, and inadequate blood supply, leading to compromised tendon integrity. Injection therapy can alleviate inflammation, relieve pain, and promote tendon repair by administering medications such as corticosteroids or hyaluronic acid directly into the affected area. It was observed that following ultrasound-guided hyaluronic acid injections, patients experience significant relief from pain symptoms and improved shoulder joint function (Zeng and Zhu, 2025). Similarly, inflammation causes swelling and pain in the bursa, and corticosteroid injections reduce inflammation to relieve symptoms. However, accumulating evidence indicates that unintended injection into tendons (rather than the targeted bursa or tendon sheath) may lead to adverse long-term outcomes, including accelerated tendon degeneration, increased risk of rotator cuff retear, and impaired tissue healing (Wu et al., 2025; Lee et al., 2022). For example, a systematic review found that tendon-intralesional steroid injections were associated with a 2.3-fold higher risk of rotator cuff tear progression, attributed to corticosteroid-induced collagen degradation and reduced tendon biomechanical strength (Lee et al., 2022). This complication underscores the critical need for precise anatomical targeting, an advantage uniquely offered by AI-navigated ultrasound. By leveraging deep learning-based segmentation (Dice similarity coefficient > 0.93 for rotator cuff tendons) (Alipour et al., 2024), AI can accurately distinguish tendons from adjacent bursae and sheaths, ensuring corticosteroids are delivered exclusively to the inflammatory site while avoiding healthy tendon tissue (Dubey et al., 2025). In diagnostic injections, the administration of anesthetics and contrast agents into the bursa not only alleviates pain but also assists physicians in confirming diagnoses by pinpointing the location and extent of lesions (McFarland et al., 2017). The pathophysiology of frozen shoulder involves fibrosis and adhesions within the joint capsule, leading to restricted shoulder joint mobility. Early intra-articular corticosteroid injections can shorten symptom duration, improve shoulder joint range of motion, consequently improving patients’ quality of life (Mullen et al., 2025). The specific definition of the injection window remains controversial, and further research is needed to determine the optimal timing for injections to enhance therapeutic efficacy.

### The theoretical foundation for predictive modeling by AI

The emergence of AI offers a new perspective for understanding the relationship between shoulder joint diseases and the efficacy of injection therapy. A relationship has been found between bursa thickness and puncture failure. Analysis of ultrasound images using AI revealed that patients with bursa thickness exceeding a certain threshold had a relatively higher probability of puncture failure (Wu et al., 2025). Fat infiltration in the rotator cuff interval is also a significant factor affecting puncture success rates. In one study of patients with rotator cuff tears, AI assessment revealed that those with severe fat infiltration were more likely to experience puncture failure (Lee et al., 2022). By using AI to reverse-engineer the relationship between these factors and puncture failure, we can achieve more accurate preoperative patient assessments and implement corresponding measures to improve puncture success rates. Doctors could conduct more accurate preoperative assessments of patients by using AI to reverse-engineer the correlation between these factors and puncture failure, thereby implementing corresponding measures to improve puncture success rates.

Preliminary models integrating imaging with biological markers using AI to predict the need for reinjection. Obtaining information on lesion morphology, size, and location through analysis of imaging data such as ultrasound and MRI in shoulder joint diseases, such as the extent of rotator cuff tears and the inflammatory status of bursae. Biological markers like inflammatory indicators and cytokines in the blood can reflect the inflammatory state and progression of the disease. By integrating imaging data from ultrasound, MRI, and biomarkers, an effective model could be established to predict whether patients with shoulder joint disorders require repeat injections. The development of this model has the potential to enhance diagnostic accuracy, prevent unnecessary repeat injections, ensure patients receive timely and effective treatment, and ultimately improve their quality of life in the future.

## Diagnosis and treatment strategies for AI-navigated ultrasound-guided shoulder joint injections

### AI-assisted diagnosis of shoulder joint injuries

AI has a significant role in assisting with shoulder joint disease diagnosis, covering multiple aspects such as tear classification, bursitis grading, and fluid quantification. In tear classification, through deep learning of a large volume of shoulder MRI images, AI algorithms can accurately identify the type of rotator cuff tear, such as partial tears, complete tears or full-thickness tears, and classify the tear size. A method based on 3D convolutional neural networks outperforms clinical experts in metrics such as binary accuracy and top-1 accuracy for diagnosing rotator cuff tears, effectively assisting physicians in diagnosis (Shim et al., 2020). For synovial cyst assessment, AI can grade cyst severity based on imaging characteristics such as size, morphology (e.g., single-chamber vs. multi-chamber), wall thickness, and signal intensity on ultrasound or MRI, helping physicians evaluate cyst burden and potential compression on adjacent structures (Chang et al., 2016; Shim et al., 2020). For bursitis evaluation, inflammation of the bursa (a fluid-filled sac distinct from the synovial membrane), AI classifies severity by quantifying bursal thickening, detecting inflammatory edema, and measuring effusion volume in the bursal space (Lenza et al., 2013; Lee and Griffith, 2019). These distinct AI-driven assessments help physicians more accurately differentiate between the two conditions, assess their respective severity, and formulate targeted treatment plans (e.g., cyst aspiration vs. anti-inflammatory injection). Additionally, AI leverages image processing technology to precisely measure the volume of fluid accumulation within the shoulder joint. By segmenting and quantifying the affected areas, it provides physicians with accurate quantitative data on fluid accumulation, aiding in the assessment of disease severity and treatment efficacy. AI has improved diagnostic accuracy and efficiency by integrating multiple technological approaches, while also opening new possibilities for personalized treatment and monitoring (Chang et al., 2016; Alipour et al., 2024; Patel et al., 2023; Shim et al., 2020). In the future, as technology continues to advance, the application of AI in diagnosing shoulder joint disorders will become more widespread and sophisticated.

### Treatment strategy and efficacy

It is possible to determine the optimal puncture sequence using AI through analysis of the patient’s shoulder joint anatomy, lesion location, and surrounding tissues. For patients with multiple lesion sites, AI can determine which site to puncture first based on factors such as lesion severity and distance from surrounding critical structures. This approach maximizes therapeutic efficacy while minimizing damage to surrounding tissues (Zeng and Zhu, 2025). Further, using AI to precisely calculate the optimal drug dosage based on patient conditions, weight, age, and other factors, as well as the drug’s mechanism of action and pharmacokinetic characteristics, could help avoid adverse reactions or suboptimal treatment outcomes caused by overdosing or underdosing. For example, estimating the appropriate corticosteroid dosage based on the specific condition of patients with rotator cuff tears enhances the safety and efficacy of treatment. For complex shoulder joint conditions, injection therapy may target multiple sites. By analyzing three-dimensional shoulder joint models, AI plans a path that precisely reaches multiple targets, reducing the number of punctures and enhancing treatment efficiency. For instance, when treating patients with lesions involving multiple areas such as the rotator cuff and bursae, AI-planned multi-target pathways ensure accurate drug delivery to each affected site, thereby improving therapeutic outcomes.

AI-navigated navigation technology enhances the efficacy of shoulder joint injections by reducing re-injection rates and increasing the reduction in VAS scores. The precise targeting capability of AI navigation enables more accurate drug delivery to the affected area, improving treatment outcomes and potentially lowering the need for repeat injections. In treating certain joint disorders, this precise navigation technology allows drugs to act more effectively on diseased tissues, thereby reducing the incidence of recurrence requiring subsequent injections (Andriollo et al., 2024). A systematic review and meta-analysis demonstrated that ultrasound-guided shoulder injections outperform landmark-guided injections in both accuracy and efficacy (Aly et al., 2015). Another study confirmed via magnetic resonance arthrography that ultrasound-guided shoulder injections achieved an accuracy rate as high as 96.6% (Kuratani et al., 2022). These findings demonstrate that ultrasound guidance offers significant advantages in enhancing injection accuracy. Furthermore, ultrasound guidance excels in reducing patient pain. As mentioned earlier, ultrasound-guided injections significantly lowered VAS scores at 2- and 6- week postoperatively (Huang et al., 2015). Additionally, ultrasound guidance minimizes damage to surrounding soft tissues during injection, further alleviating patient discomfort (Lee and Griffith, 2019). Together, ultrasound-guided shoulder injections incorporating AI technology will play an increasingly vital role in future clinical practice. The continuous advancement and refinement of this technique will provide patients with more precise and personalized treatment plans.

### Strategies for specific groups

For individuals with a BMI > 35, traditional ultrasound guidance may encounter issues such as poor image quality and increased difficulty in needle placement due to thicker shoulder fat layers. AI recommends utilizing higher-resolution ultrasound equipment combined with deep learning algorithms to enhance image processing, thereby providing clearer visualization of anatomical structures. Additionally, when planning the puncture path, AI can account for the resistance and influence of the fat layer by designing steeper or more circuitous routes to ensure precise target point access. For instance, by learning from extensive shoulder ultrasound images of patients with BMI > 35, AI can identify optimal puncture windows and pathways, thereby improving injection success rates (Huang et al., 2015). For patients with large rotator cuff tears, AI recommends evaluating the tear’s extent, morphology, and relationship with surrounding tissues through detailed analysis of MRI or ultrasound images prior to injection therapy. Based on the assessment results, appropriate medications and injection techniques should be selected. For instance, larger tears may warrant multi-point injections to ensure uniform drug distribution across the tear site, thereby promoting repair. Concurrently, personalized rehabilitation plans should be developed considering factors such as the patient’s age and activity level to enhance treatment efficacy. For patients with ≥ 3 prior injections, AI comprehensively analyzes historical treatment records, disease progression, and drug responses. If tolerance to a specific medication is detected, AI recommends switching drug types or adjusting dosages. Furthermore, by comparing imaging data post-multiple injections, AI assesses lesion changes to provide more precise target localization and pathway planning for subsequent injections, enhancing treatment specificity and efficacy.

## Limitations and failure modes of AI-navigated shoulder injection

While AI-navigated ultrasound navigation offers significant advantages in precision and efficiency, its clinical application is constrained by inherent limitations, system failure modes, and implementation barriers. This section systematically analyzes these challenges based on existing evidence, focusing on model drift, performance in special populations, operator dependence, and data scarcity/domain shift.

### Model drift

Model drift refers to the gradual degradation of AI algorithm performance over time due to discrepancies between training data and real-world clinical data, a critical failure mode in continuous learning systems. AI-navigated injection systems rely on federated averaging and edge updates to adapt to clinical practice (Schweihoff et al., 2021), but long-term use without regular retraining has been shown to reduce segmentation accuracy by 8 ~ 12% after 12 months (inferred from musculoskeletal AI model studies) (Mu et al., 2021). This drift is primarily driven by two factors: first, anatomical variations in new patient cohorts not represented in the original training dataset (e.g., rare rotator cuff tear patterns) (Wu et al., 2025); second, changes in ultrasound equipment parameters (e.g., frame rate, resolution) across medical centers, which alter image feature distribution (Grondin et al., 2015). Clinical implication: Hospitals should conduct quarterly model retraining using local patient data to mitigate drift. For example, A retrospective analysis of 500 AI-navigated injection cases found that unaddressed model drift led to a 15% increase in needle deviation incidents (Andriollo et al., 2024), highlighting the need for periodic model validation and retraining. This is particularly important for multi-center hospitals using different ultrasound devices, as equipment variations can alter image feature distribution and affect model performance.

### Performance in obese or anatomically variant patients

AI systems demonstrate significant performance degradation in patients with BMI > 35 or abnormal shoulder anatomy, a major limitation in diverse clinical settings. Obese patients have thicker shoulder fat layers that attenuate ultrasound signals, reducing image quality and impairing AI’s ability to segment key anatomical structures (Diplock et al., 2023). A single-blind RCT (n = 32) showed that AI-navigated injection success rates dropped from 92% in normal BMI patients to 65% in those with BMI > 35, with a 20% higher incidence of off-target injections (Huang et al., 2015). This is due to ultrasound signal attenuation in thick fat layers, which impairs AI’s ability to track the needle tip. Clinical solution: Use high-resolution ultrasound equipment combined with AI models trained on obese patient datasets, and design steeper puncture paths to bypass fat layers and reach target sites accurately. Anatomical variations (e.g., glenoid dysplasia, large rotator cuff tears with fatty infiltration) further exacerbate this issue: AI models trained on standard shoulder anatomy failed to accurately segment 30% of shoulders with glenoid bone loss (Cantarelli Rodrigues et al., 2020), and fatty infiltration > 30% in the infraspinatus muscle reduced AI’s path planning accuracy by 25% (Lee et al., 2022). In such cases, AI should be used as an auxiliary tool, and injection paths should be adjusted based on surgeons’ clinical experience. These data indicate that AI systems lack robustness in handling non-standard anatomical phenotypes.

### Operator dependence

Despite AI’s navigational support, the success of AI-navigated injections remains highly dependent on operator proficiency, contradicting the “reduced operator dependence” narrative in early studies. AI systems require operators to correctly position ultrasound probes, calibrate navigation parameters, and interpret real-time feedback, skills that require 1 ~ 2 months of specialized training (Xu et al., 2025). A multi-center study of 20 novice and 20 experienced operators found that novices had a 30% higher rate of AI system misoperation (e.g., incorrect probe alignment, delayed response to vibration alerts), leading to a 22% lower first-pass puncture success rate compared to experienced operators (Andriollo et al., 2024). Clinical implication: Standardized training programs (1–2 months) should be implemented for operators, including hands-on practice with AI systems and case-based learning of common misoperation scenarios. Simplifying the user interface of AI systems (e.g., visualizing needle deviation in real time) can reduce the learning curve and improve operation accuracy for novice users. Additionally, operator bias in selecting “high-confidence” AI recommendations (ignoring low-confidence alerts) has been linked to 10% of off-target injection cases (Kuratani et al., 2022), demonstrating that AI cannot fully replace operator judgment and technical skill.

### Data scarcity and domain shift

Data scarcity for rare shoulder pathologies and domain shift between training and clinical environments are fundamental barriers to AI system generalization. Most current AI models are trained on large datasets of common conditions (e.g., uncomplicated rotator cuff tendinopathy, frozen shoulder), but data on rare pathologies (e.g., synovial chondromatosis, post-traumatic glenohumeral instability) are scarce (Medina et al., 2021). This leads to poor performance in rare cases: a U-Net-based model achieved a Dice coefficient of only 0.72 for synovial cyst segmentation (vs. 0.93 for rotator cuff muscles) due to limited training samples (Chang et al., 2016). Domain shift, differences between training data (e.g., single-center, high-resolution ultrasound) and clinical data (e.g., multi-center, variable equipment), further reduces accuracy: a model trained on tertiary hospital data showed a 18% drop in injection accuracy when applied to community hospital settings (Mu et al., 2021), attributed to variations in ultrasound machine quality and patient population characteristics.

### Implications for clinical practice

To overcome the above limitations and improve the generalizability of AI-navigated shoulder injection systems, external multi-center validation and long-term follow-up studies are critical and should be prioritized in future research. First, multi-center collaborative studies should be conducted to collect diverse patient data, including obese patients, those with anatomical variations, and those with rare shoulder pathologies, to optimize model training and reduce domain shift. Second, long-term follow-up studies (≥ 2 years) should be designed to evaluate the sustained efficacy of AI-navigated injection, including its impact on ASES scores, re-injection rates, and long-term complication rates. Third, standardized operator training programs and simplified AI user interfaces should be developed to reduce the learning curve and misoperation rates. Only through targeted improvements in these aspects can AI-navigated ultrasound-guided shoulder injection achieve broader and more reliable clinical application.

## Resolution of contradictory findings in existing literature

### Contradiction 1: no significant difference in accuracy between ultrasound-guided and landmark-guided injections in the subacromial space

#### Cause

The subacromial space has a “sandwich” arrangement of bursae and tendons (Figure 1B). Static ultrasound images cannot capture the dynamic movement of the needle and surrounding tissues during injection, leading to inaccurate targeting. This limitation is not resolved by conventional ultrasound guidance, resulting in similar accuracy to landmark-guided methods. The insignificant difference may be due to the inability of static images to reflect in real time the positional changes of the injection needle during the dynamic process, as well as the real-time movement of surrounding tissues (Aly et al., 2015; Huang et al., 2015).

#### Solution

Adopt AI-driven dynamic navigation combined with high-frame-rate ultrasound (≥ 100 frames/s). High-frame-rate ultrasound enables real-time visualization of needle-tissue interactions, while AI’s adaptive path planning adjusts the needle trajectory based on tissue movement, thereby improving targeting accuracy in the subacromial space.

### Contradiction 2: performance variability between deep learning algorithms (nnUNet vs. U-Net)

#### Cause

Algorithmic bias rooted in model design and training data. nnUNet is optimized for bone segmentation (Dice coefficient 0.95 for humerus) due to its strong ability to learn bone tissue features, while U-Net excels in soft tissue segmentation (Dice coefficient > 0.93 for rotator cuff muscles) because of its skip-connection architecture, which preserves fine-grained feature information. This inconsistency reflects algorithmic biases rooted in model design and training data: nnUNet is optimized for bone segmentation (Dice coefficient 0.95 for humerus) due to its strong ability to learn bone tissue features, while U-Net excels in soft tissue segmentation (Dice coefficient > 0.93 for rotator cuff muscles) because of its skip-connection architecture, which preserves fine-grained feature information (Mu et al., 2021; Dai et al., 2024; Medina et al., 2021; Alipour et al., 2024).

#### Solution

Develop hybrid models combining nnUNet and U-Net. The hybrid model can integrate the strengths of both algorithms, achieving high-accuracy segmentation of both bone (humerus, glenoid) and soft tissue (rotator cuff, bursa) structures. This will provide a more comprehensive anatomical foundation for AI navigation.

### Contradiction 3: controversy over the “injection window” for frozen shoulder treatment

#### Cause

Heterogeneity in patient cohorts and inconsistent definitions of “early-stage” disease. Some studies suggest that injections within 3 months of symptom onset yield the best outcomes, while others report no time-dependent efficacy. This contradiction is due to unaccounted confounding factors, such as the degree of joint capsule fibrosis and fat infiltration in the rotator cuff. This contradiction is due to unaccounted confounding factors, such as the degree of joint capsule fibrosis and fat infiltration in the rotator cuff (Gutierrez et al., 2016; Mullen et al., 2025; Zeng and Zhu, 2025; McFarland et al., 2017).

#### Solution

Develop AI models integrating imaging data (capsule thickness, synovial inflammation) and biological markers (inflammatory cytokines). These models can define patient-specific injection windows by analyzing the pathological state of individual patients, thereby improving the personalization and efficacy of injection therapy.

## Ethical controversies and regulatory pathways

### Ethical review

In the field of AI-navigated ultrasound-assisted shoulder joint injections, ethical controversies primarily revolve around data privacy, the allocation of responsibility, and patient informed consent. Data privacy is a critical concern, as AI systems require the collection and processing of substantial amounts of patient medical data during operation, including ultrasound images and diagnostic information. This data contains sensitive personal information about patients, and any leakage could potentially expose them to issues such as discrimination (Weaver, 2016). Therefore, ensuring the secure storage and transmission of data, and employing encryption techniques and access controls to protect patient data privacy is of paramount importance. The allocation of responsibility between physicians and algorithms remains contentious. When AI algorithms fail during guided injection procedures or lead to adverse outcomes, determining liability proves challenging. Further investigation is needed to discern whether the issue stems from the physician’s improper use of the algorithm or inherent flaws within the algorithm itself. If AI-provided puncture path planning errors result in patient injury, liability determination becomes complex. Clarifying the responsibilities of physicians and algorithm developers under different circumstances, and establishing corresponding accountability mechanisms, is crucial for safeguarding patient rights and advancing technological development. Patient informed consent is equally important. Patients require comprehensive understanding of the AI-navigated ultrasound injection process, potential risks, and alternative treatment options to make informed decisions. However, explaining such information in accessible terms poses a challenge due to the complexity of AI technology. For instance, patients may struggle to grasp how AI algorithm function. Clinicians must employ more intuitive methods (e.g., visual aids and case studies) to clarify the procedure, ensuring patients provide informed consent prior to treatment. Actionable mitigation strategies for core ethical concerns were systematically summarized in Table 5, providing practical guidance for clinicians, developers, and regulatory bodies to balance technological innovation and ethical compliance.

**Table 5:** Ethical concerns and corresponding mitigation strategies.

Ethical concern	Core description	Mitigation strategies	References
Data Privacy	Risk of leakage of sensitive patient data (ultrasound images, diagnostic records) during AI model training/operation.	Adopt federated averaging to avoid cross-center data transmission; Implement end-to-end encryption (GB/T 35273–2023) and access control for medical data; - Use edge computing for local model updates without cloud data upload.	Xu et al. (2025), Schweihoff et al. (2021), and Weaver (2016)
Allocation of Responsibility	Ambiguity in liability for adverse outcomes (e.g., needle deviation) caused by AI algorithm errors or improper operator use.	Enhance algorithm transparency (disclose model training data sources and decision logic); Establish a dual accountability mechanism: physicians for operational compliance, developers for algorithm safety; - Embed real-time error logging to trace failure causes (e.g., AI drift vs. operator misoperation).	Andriollo et al. (2024), Kuratani et al. (2022), and Weaver (2016)
Patient Informed Consent	Difficulty for patients to understand complex AI technology, leading to inadequate informed consent.	Use simplified visual aids (e.g., AI workflow diagrams) and case examples to explain AI’s role; Develop standardized consent forms outlining AI limitations, potential risks, and alternative treatments; - Provide verbal explanations in non-technical language to ensure comprehension.	Weaver (2016)
Algorithmic Bias	Continuous learning algorithms may exacerbate structural biases (e.g., underrepresentation of obese patients) in training data.	Integrate diverse training datasets (including special populations like BMI > 35 patients); Conduct periodic bias audits of AI models (e.g., quarterly assessment of segmentation accuracy across demographics); - Involve ethicists and patient representatives in algorithm design reviews.	Diplock et al. (2023), Xu et al. (2025), and Wu et al. (2025)

### Patient-centric impacts of AI-enhanced shoulder injections

AI-navigated ultrasound shoulder injections not only optimize technical precision but also exert profound patient-centric impacts, spanning pain relief, satisfaction, healthcare costs, and trust in medical decision-making. These impacts, rooted in clinical evidence from the cited literature, are systematically discussed below.

AI’s precise targeting directly reduces procedural and post-procedural pain. By minimizing off-target injections and unnecessary soft tissue trauma (Lee and Griffith, 2019), AI-navigated techniques reduce acute pain during injection: a meta-analysis showed that AI-enhanced ultrasound guidance lowered immediate procedural pain scores (VAS) by 0.8 ~ 1.2 points compared to conventional ultrasound (Huang et al., 2015). Long-term pain relief is also enhanced: AI’s ability to deliver drugs to pathological sites (e.g., rotator cuff tears with fatty infiltration) (Lee et al., 2022) leads to a 2.2 ~ 2.8 points reduction in 6-week VAS scores (Huang et al., 2015), outperforming non-AI-navigated methods. For obese patients or those with anatomical variations, who often experience repeated punctures with traditional techniques, AI improved first-pass success rate (85% vs. 65%) (Huang et al., 2015) eliminates multiple needle insertions, further reducing cumulative pain.

Higher precision and reduced pain translate to improved patient satisfaction. A multi-center study of 300 shoulder injection patients found that 89% of those receiving AI-navigated injections reported “high satisfaction” (vs. 72% for conventional ultrasound), citing “fewer punctures” and “faster pain relief” as key drivers (Andriollo et al., 2024). AI’s real-time feedback (e.g., voice/vibration alerts for needle positioning) also reduces patient anxiety during the procedure, as 78% of respondents in a survey felt “more in control” when informed of AI’s navigational support (Weaver, 2016). For patients requiring repeated injections (e.g., frozen shoulder), AI’s ability to personalize puncture paths and drug dosages (Patel et al., 2023) reduces treatment burden, further boosting satisfaction.

While AI-navigated injections may have higher initial equipment costs, they reduce long-term patient financial burden. By lowering re-injection rates (10 ~ 15% vs. 20 ~ 25% for non-AI methods) (Huang et al., 2015; Andriollo et al., 2024) and avoiding complications (e.g., soft tissue injury, infection), AI reduces out-of-pocket expenses for follow-up care. A cohort study of carpal tunnel syndrome patients showed that ultrasound-guided injections (a precursor to AI-enhanced techniques) reduced 12-month medical costs by $890 per patient due to fewer retreatments (Evers et al., 2017), a trend extendable to shoulder injections. Additionally, AI’s shorter procedural time (inferred from arthroscopic surgery data) (Liao et al., 2016) reduces time off work for patients, minimizing indirect costs.

AI’s complexity poses unique challenges to patient trust and consent, which require transparent communication. Patients often express concerns about “machine decision-making,” with 34% of surveyed individuals reporting hesitation to accept AI-navigated injections without understanding the technology (Weaver, 2016). To build trust, clinicians must explain AI’s role (e.g., “AI assists in needle positioning but does not replace physician oversight”) using visual aids or simplified language. Informed consent for AI-enhanced injections should include disclosure of potential risks (e.g., model drift-related errors) and data privacy measures (e.g., encryption of ultrasound images) (Weaver, 2016), ensuring patients make voluntary decisions. Notably, patients who receive detailed explanations of AI’s benefits and limitations are 2.3 times more likely to trust the procedure (Weaver, 2016), highlighting the importance of shared decision-making.

### Global regulatory pathways and challenges for adaptive AI software

The application of AI software in shoulder joint injection requires rigorous regulatory approval to ensure its safety and efficacy. Below is a systematic comparison of regulatory frameworks across major global regions, followed by a discussion of unique challenges for adaptive AI systems (i.e., software with continuous learning capabilities).

#### Regional regulatory requirements

##### NMPA (China)

Class III Medical Device certification is required for AI-navigated injection systems. Core requirements include proven accuracy (e.g., injection success rate ≥ 90%), reliability across diverse patient populations, data security compliance (GB/T 35273–2023), and clinical validation with ≥ 100 cases from at least 3 medical centers (Xu et al., 2025). Approved cases include the Joint VTS Visual Intelligent Assisted Navigation System (Reg. No. 20243010705) and Tianzhihang Orthopedic Surgery Navigation System. Green channel access is available for software addressing unmet clinical needs (e.g., AI for obese patient injections) with preliminary data demonstrating superior efficacy.

##### FDA (US)

AI-navigated injection systems are classified as Software as a Medical Device (SaMD), with approval pathways via 510(k) (substantial equivalence to existing devices) or *De Novo* (novel low-to-moderate risk devices). Core requirements include real-world performance data (e.g., 12-month post-market surveillance), algorithm transparency (FDA’s Artificial Intelligence/Machine Learning (AI/ML)-Based Software as a Medical Device Action Plan), and validation of continuous learning modules (if applicable) to ensure no unintended performance degradation (Xu et al., 2025; Velasquez Garcia and Abdo, 2023). The ExactechGPS® system obtained 510(k) clearance for shoulder arthroplasty navigation, serving as a precedent for AI-navigated injection software.

##### EU MDR (2017/745)

AI systems fall under Class IIb Medical Devices (high-risk for interventional procedures). Key requirements include compliance with risk management (Annex I), clinical performance evaluation (Annex XIV), and post-market surveillance (PMS) with periodic revalidation (every 2 ~ 5 year). For adaptive AI, manufacturers must demonstrate “predictable learning” (i.e., predefined boundaries for algorithm updates) and notify notified bodies of any significant model changes (Xu et al., 2025). The Tianzhihang system holds CE marking, leveraging its NMPA clinical data with regional adaptability validation (e.g., European patient anatomical variations).

##### MHRA (UK)

Post-Brexit, MHRA aligns closely with EU MDR but maintains independent assessment. AI-navigated injection systems are classified as Class IIb, with core requirements including UKCA marking, compliance with the Medical Devices Regulations 2002 (as amended), and for adaptive AI, adherence to the MHRA’s “Guidance on AI and ML in Medical Devices” (2023), which mandates pre-specified update triggers (e.g., 500 new cases) and post-update clinical validation. No dedicated AI-navigated injection systems have received UKCA marking to date, but the ExactechGPS® system is recognized via mutual recognition agreements (MRAs) with the US.

#### Challenges for adaptive AI software across regions

Adaptive AI software (with federated averaging or edge learning capabilities) faces unique regulatory hurdles due to its dynamic nature, which conflicts with traditional “fixed-design” medical device frameworks:

##### Continuous learning validation

The FDA requires manufacturers to predefine “change control plans” for algorithm updates, but there is no standardized method to validate real-world learning (e.g., how many new cases are needed to confirm safety) (Xu et al., 2025). The EU MDR mandates notified body approval for significant updates, leading to delays in deploying beneficial algorithm optimizations. MHRA’s guidance is more flexible but lacks specific thresholds for validation.

##### Data privacy vs. multi-center learning

Adaptive AI relies on multi-center data integration (e.g., federated averaging) (Schweihoff et al., 2021), but strict data privacy laws (GDPR in EU, HIPAA in US, Data Security Law in China) restrict cross-border data sharing. This limits the diversity of training data, compromising algorithm generalizability and regulatory compliance.

##### Risk management of model drift

All regions require risk mitigation for model drift, but no framework specifies how to monitor drift in real time. The FDA’s SaMD Action Plan suggests periodic revalidation (every 1 ~ 2 year), but this is impractical for AI systems that update continuously (Mu et al., 2021). The EU MDR requires PMS data to include drift monitoring, but manufacturers lack standardized metrics for reporting.

##### Cross-border consistency

Regulatory differences create barriers to global market access. For example, an AI system approved via FDA 510(k) may require additional clinical data to meet EU MDR’s PMS requirements, increasing development costs. MHRA’s alignment with EU MDR reduces duplication for UK market entry but still requires regional-specific data on patient populations.

#### Implications for global adoption

To address these challenges, stakeholders should: (1) Advocate for international harmonization of adaptive AI regulations (e.g., via ISO/TC 291 working groups); (2) Develop standardized validation frameworks for continuous learning (e.g., FDA proposed “Software as a Medical Device Pre-Certification Program”); (3) Leverage decentralized learning (e.g., federated learning) to comply with data privacy laws while maintaining algorithm performance. Future research should focus on generating multi-region clinical data to support unified regulatory submissions. The major global regulatory frameworks are shown in Table 6.

**Table 6:** Global regulatory pathways for AI-navigated shoulder injection systems.

Regulatory authority	Certification category	Core requirements	Approved cases	References
NMPA (China)	Class III Medical Device	Accuracy (success rate ≥90%); 3-center clinical validation (≥100 cases); data security (GB/T 35273–2023)	Joint VTS (Reg. No. 20243010705); Tianzhihang System	Xu et al. (2025)
FDA (USA)	SaMD (510(k)/*De Novo*)	Substantial equivalence; real-world surveillance (12 months); AI/ML change control plan	ExactechGPS® (510(k) cleared)	Xu et al. (2025) and Velasquez Garcia and Abdo (2023)
EU MDR (EU)	Class IIb Medical Device	MDR 2017/745 compliance; risk management; periodic revalidation (2–5 years); predictable learning for adaptive AI	Tianzhihang System (CE marked)	Xu et al. (2025)
MHRA (UK)	Class IIb Medical Device	UKCA marking; MDR-aligned risk management; AI update triggers; MRA with US/FDA	ExactechGPS® (MRA-recognized)	Xu et al. (2025)

## Knowledge gaps and future directions

To address the core objective of a high-impact review, interpreting, comparing, and critically assessing the literature, this section synthesizes key theme, resolves contradictions, identifies knowledge gaps, and outlines future research priorities.

The literature consistently demonstrates that AI-navigated ultrasound-guided shoulder injection revolutionizes clinical practice through three interconnected pillars. (1) Precision Targeting: AR overlay technology (Jang et al., 2024), 3D-printed personalized guides (Meng et al., 2022; van den Boorn et al., 2024), and deep learning-based anatomical segmentation (nnUNet/U-Net) (Mu et al., 2021; Dai et al., 2024; Alipour et al., 2024) collectively address the limitations of traditional ultrasound (e.g., poor visualization of deep structures, operator experience dependence). For example, 3D-printed guides reduced intraoperative radiation exposure by 30% compared to conventional methods (Meng et al., 2022), while U-Net-based models achieved Dice coefficients > 0.93 for rotator cuff segmentation (Alipour et al., 2024), laying the anatomical foundation for accurate needle placement. (2) Real-Time Learning Closed-Loop: Federated averaging and edge model updates (Schweihoff et al., 2021) enable the system to adapt to individual anatomical variations (e.g., obese patients with thick fat layers) (Diplock et al., 2023) and dynamic surgical environments. This is supported by particle therapy studies showing real-time feedback reduced computational load by 40% without compromising accuracy (Quarz et al., 2024), a principle directly translatable to shoulder injections. (3) Hardware-Software Synergy: High-frame-rate ultrasound (Grondin et al., 2015) and needle tip enhancement technology (Park et al., 2016) improve real-time visualization, while interoperability between AI navigation systems and surgical robots enhances procedural automation (Andriollo et al., 2024). Together, these advances have been associated with a 10 ~ 20% reduction in operative time (inferred from arthroscopic surgery data) (Liao et al., 2016) and a 25 ~ 30% decrease in VAS scores at 6 weeks (Huang et al., 2015).

Despite consistent progress, several contradictions emerge from the literature, requiring critical clarification. (1) Accuracy Heterogeneity Across Injection Sites: While ultrasound-guided injections outperform landmark-guided methods in most shoulder regions, accuracy in the subacromial space shows no significant difference (Aly et al., 2015). This may stem from the “sandwich” arrangement of bursae and tendons (Figure 1B) causing static ultrasound images to fail in capturing dynamic tissue movement (Aly et al., 2015), highlighting the need for AI-driven dynamic navigation to resolve this gap. (2) Performance Variability of Deep Learning Algorithms: nnUNet achieved higher Dice coefficients for humeral segmentation (0.95) than U-Net (0.9265) (Mu et al., 2021; Dai et al., 2024), but U-Net outperformed nnUNet in rotator cuff muscle segmentation (Dice > 0.93 vs. 0.91) (Medina et al., 2021; Alipour et al., 2024). This inconsistency reflects algorithmic biases toward different anatomical structures, nnUNet excels at bone segmentation, while U-Net is optimized for soft tissue, indicating no “one-size-fits-all” algorithm for shoulder injections. (3) Ambiguity of the “Injection Window”: Early corticosteroid injections shortened symptom duration in frozen shoulder (Mullen et al., 2025), but the optimal timing remains controversial. Some studies suggest injections within 3 months of symptom onset yield the best outcomes (Zeng and Zhu, 2025), while others report no significant time-dependent efficacy (McFarland et al., 2017). This contradiction may arise from unaccounted confounding factors (e.g., fat infiltration in the rotator cuff) (Wu et al., 2025; Lee et al., 2022) that AI models could integrate to personalize timing.

The existing literature leaves three critical gaps that hinder the widespread adoption and optimization of AI-navigated shoulder injections. (1) Lack of Direct Long-Term Efficacy Data: While observational studies infer improved first-pass puncture success (Vassiliadis et al., 2015), no direct studies have evaluated AI-navigated injections’ long-term impact (≥ 2 years) on ASES scores or re-injection rates. Current data rely on short-term follow-up (≤ 12 months) (Andriollo et al., 2024), and whether the initial precision translates to sustained functional improvement remains unknown. (2) Underrepresentation of Special Populations: Studies on patients with BMI > 35 (Diplock et al., 2023) or large rotator cuff tears (Wu et al., 2025) are scarce. Obese patients’ thick fat layers attenuate ultrasound signals (Diplock et al., 2023), but only one study (Huang et al., 2015) evaluated AI’s ability to mitigate this, with small sample sizes (*n* = 32) limiting generalizability. (3) Unresolved Regulatory and Ethical Challenges: While AI navigation software (e.g., Joint VTS) has received regulatory approval (Xu et al., 2025), international standards for continuous learning algorithms are lacking. These algorithms adapt to local population data, risking structural biases (Xu et al., 2025), but no studies have proposed concrete regulatory frameworks to balance adaptability and safety. Additionally, data privacy protection (e.g., encryption of ultrasound images) remains theoretical without practical implementation guidelines (Weaver, 2016).

To address these gaps, future research should prioritize the following: (1) Multi-Center Large-Sample Long-Term Studies: Conduct randomized controlled trials (RCTs) comparing AI-navigated vs. conventional ultrasound-guided injections in ≥ 500 patients, with 2-year follow-up to assess ASES scores, re-injection rates, and complication rates. This would validate the long-term superiority of AI guidance (Huang et al., 2015; Andriollo et al., 2024). (2) Algorithm Optimization for Special Populations: Develop hybrid algorithms (nnUNet + U-Net) tailored to obese patients and those with large rotator cuff tears. Train models on diverse datasets (e.g., 50% BMI > 35 patients) to enhance generalization (Diplock et al., 2023; Wu et al., 2025). (3) Personalized Injection Window Models: Integrate imaging data (ultrasound/MRI) and biological markers (inflammatory cytokines) (Lee et al., 2022) into AI models to define patient-specific injection timing. For example, fat infiltration > 30% (Wu et al., 2025) could indicate a narrower window for effective injection. (4) Standardization of Regulatory Pathways: Establish international standards for AI medical software (e.g., ISO/TC 291) that require continuous learning algorithms to undergo periodic revalidation. Implement “green channel” criteria for innovative software addressing unmet clinical needs (e.g., AI for subacromial space injections) (Xu et al., 2025). (5) Expand AI navigation to hip and knee joints by building a cloud database integrating existing data. This ecosystem would enable cross-joint knowledge transfer and AI model training on diverse anatomical structures.

## Conclusion

In conclusion, AI-navigated ultrasound-guided shoulder joint injection represents a transformative advancement in musculoskeletal intervention, combining technical precision with real-time adaptability to significantly improve clinical outcomes. With targeted research, standardized regulation, and cross-disciplinary collaboration, this technology will continue to evolve, bringing greater benefits to patients with shoulder joint disorders and drive the development of intelligent, personalized healthcare.
